# Immune-inducible non-coding RNA molecule *lincRNA-IBIN* connects immunity and metabolism in *Drosophila melanogaster*

**DOI:** 10.1371/journal.ppat.1007504

**Published:** 2019-01-11

**Authors:** Susanna Valanne, Tiina S. Salminen, Mirva Järvelä-Stölting, Laura Vesala, Mika Rämet

**Affiliations:** 1 Laboratory of Experimental Immunology, BioMediTech Institute and Faculty of Medicine and Life Sciences, University of Tampere, Tampere, Finland; 2 PEDEGO Research Unit, and Medical Research Center Oulu, University of Oulu, and Department of Children and Adolescents, Oulu University Hospital, Oulu, Finland; 3 Department of Pediatrics, Tampere University Hospital, Tampere, Finland; University of Oxford, UNITED KINGDOM

## Abstract

Non-coding RNAs have important roles in regulating physiology, including immunity. Here, we performed transcriptome profiling of immune-responsive genes in *Drosophila melanogaster* during a Gram-positive bacterial infection, concentrating on long non-coding RNA (lncRNA) genes. The gene most highly induced by a *Micrococcus luteus* infection was *CR44404*, named *Induced by Infection* (*lincRNA-IBIN*). *lincRNA-IBIN* is induced by both Gram-positive and Gram-negative bacteria in *Drosophila* adults and parasitoid wasp *Leptopilina boulardi* in *Drosophila* larvae, as well as by the activation of the Toll or the Imd pathway in unchallenged flies. We show that upon infection, *lincRNA-IBIN* is expressed in the fat body, in hemocytes and in the gut, and its expression is regulated by NF-κB signaling and the chromatin modeling brahma complex. In the fat body, overexpression of *lincRNA-IBIN* affected the expression of Toll pathway -mediated genes. Notably, overexpression of *lincRNA-IBIN* in unchallenged flies elevated sugar levels in the hemolymph by enhancing the expression of genes important for glucose retrieval. These data show that lncRNA genes play a role in *Drosophila* immunity and indicate that *lincRNA-IBIN* acts as a link between innate immune responses and metabolism.

## Author summary

*Drosophila melanogaster* is a powerful genetic model for studying the innate immune mechanisms conserved from flies to humans. With recent methodology, such as whole transcriptome analyses, novel non-protein coding genes in addition to protein coding genes are being increasingly identified. These long and short non-coding RNA genes are located between and within protein coding genes in the genome, and their functions are largely uncharacterized. In humans, such RNA genes have been shown to affect numerous physiological processes including immune responses. In *Drosophila*, very few non-coding RNA genes have so far been characterized in detail. In this study, we have identified and characterized an immune-inducible long non-coding RNA gene, *lincRNA-IBIN*. *lincRNA-IBIN* is induced by exposure to bacteria as well as the parasitoid wasp, *Leptopilina boulardi*, suggesting a general role in humoral and cellular innate immunity. Accordingly, forced expression of *lincRNA-IBIN* enhances the expression of genes involved in carbohydrate catabolism and elevates hemolymph glucose levels in *Drosophila*. These results indicate that *lincRNA-IBIN* acts as a link between immunity and metabolism in *Drosophila*. As research in *Drosophila* has often resulted in the identification of evolutionarily conserved mechanisms also in mammals, it remains to be studied whether long non-coding RNA genes regulate metabolism upon an infection also in humans.

## Introduction

The fruit fly *Drosophila melanogaster* (D. melanogaster) is a widely used model system in immunological studies [1]. *Drosophila* has an elegant innate immune response that includes both the cellular and the humoral arms [2,3]. Activation of the cellular immune response involves mechanisms such as recognition, phagocytosis, encapsulation and the killing of parasites [4,5]. The humoral immune response is based on microbial recognition primarily by peptidoglycan recognition proteins leading to the production of antimicrobial peptides (AMPs)[6–9]. The humoral immune response is mainly mediated by two evolutionarily conserved NF-κB signaling pathways, the Toll and the Immune deficiency (Imd) pathway [10–12].

Recently, it has become evident that beside the protein coding genes that positively or negatively regulate the humoral and cellular innate immune responses, there is a multitude of short and long non-coding RNA genes that affect innate immune responses [13–16]. In between and within protein coding genes in the genome, there are thousands of uncharacterized non-coding RNA genes. Small non-coding RNAs (<200 nucleotides) are considered to have more of a “housekeeping RNA” role. However, the functions of long non-coding RNA (lncRNA, >200 nucleotides) genes are more diverse [17]. Although the number of lncRNAs is still a matter of debate, recent meta-analyses posit the human genome to give rise to >60,000 lncRNAs, albeit the majority is probably expressed at low levels [18,19]. In fruit flies, there are fewer lncRNAs in the genome and the ratio of lncRNAs to protein coding genes is lower than in humans [20]. The current lncRNA numbers can be found in the NONCODE Version v5.0 database (www.noncode.org).

The expression patterns of lncRNAs are highly specific to tissue, developmental stage and environmental conditions (reviewed in [14,15]) and they are thought to have tightly controlled biological roles. Recent studies have indicated that lncRNAs play an important functional role in innate immune responses, and specifically in innate immune cells. In mammals, lncRNA genes are expressed in monocytes, macrophages, dendritic cells, neutrophils, T-cells and B-cells [13]. A growing list of lncRNA genes, for example *LincRNA-Cox2* [21], *Lethe* [22], *PACER* [23] and *TNFα regulating hnRNPL interacting lncRNA* (*THRIL*)[24], has been found to control gene expression in immune cells [13].

To study the role of lncRNA genes in the *Drosophila* immune response, we performed transcriptome analysis in *D*. *melanogaster* upon a bacterial infection with the Gram-positive *Micrococcus luteus* (*M*. *luteus*), giving particular emphasis to long non-coding RNA (lncRNA) genes. The most responsive of all transcripts was the lncRNA gene *CR44404*, which was upregulated 1300-fold upon a *M*. *luteus* infection. Here, we show that *CR44404* is highly induced by both Gram-positive and Gram-negative bacteria in *Drosophila* adults and by a parasitoid wasp infection in *Drosophila* larvae. Because of the inducible nature of the *CR44404* gene, we named it *lincRNA-IBIN* (*Induced By INfection*). Finally, we show that *lincRNA-IBIN* acts as a link between innate immune responses and metabolism by modulating the expression of genes regulating carbohydrate and peptide metabolism and affecting glucose levels in the hemolymph.

## Results

### Long non-coding RNA *IBIN* (*CR44404*) expression is induced by an infection in *Drosophila*

**Fig 1:**
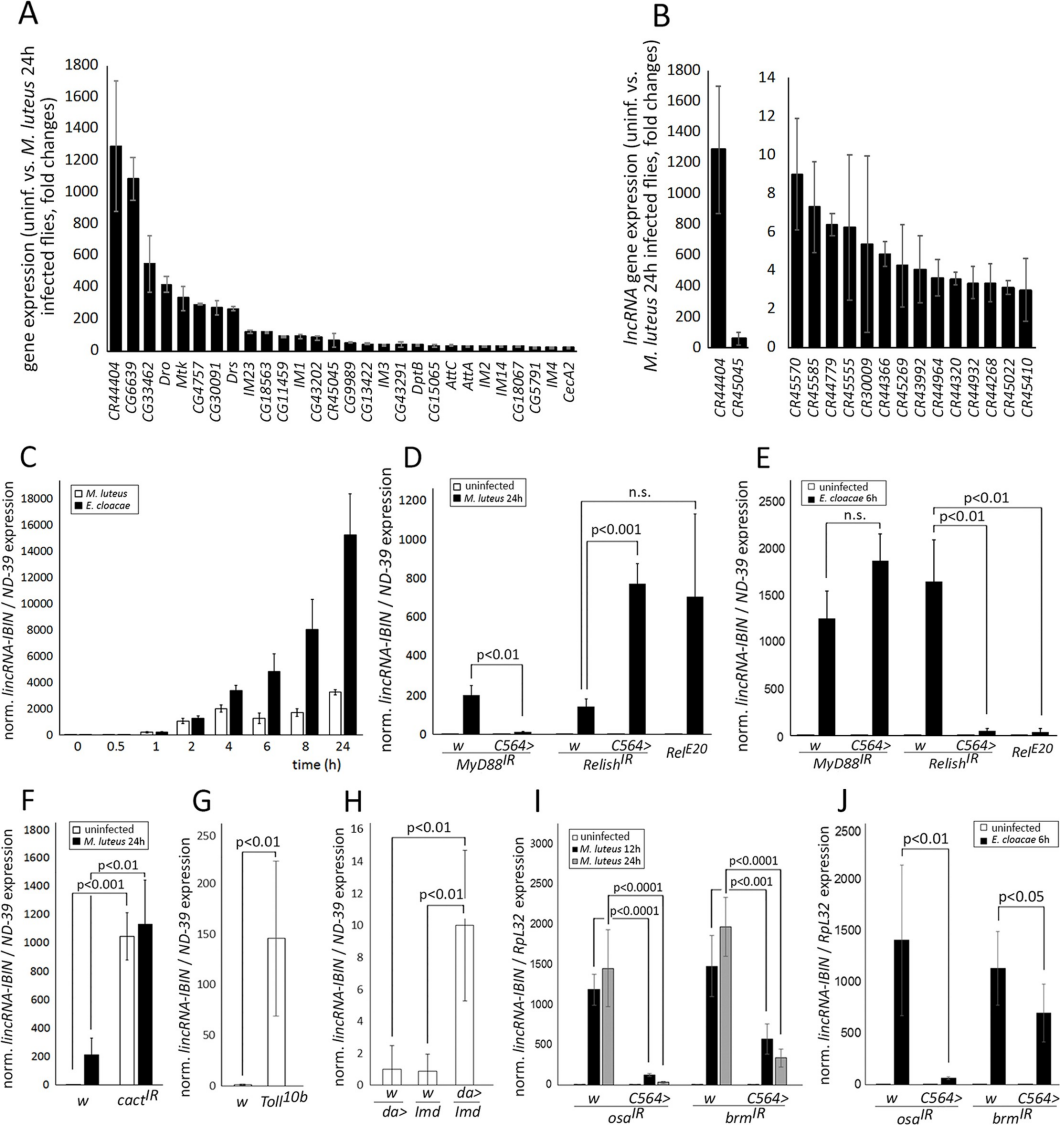
*lincRNA-IBIN* (*CR44404*) expression is strongly induced by Gram-positive and Gram-negative bacteria and its expression is regulated by the Toll and Imd pathways and a functional BAP complex. A) In a whole transcriptome analysis, 28 genes were more than 18-fold upregulated after a *M*. *luteus* infection. The highest upregulation in infected flies was found in a long non-coding RNA gene, *CR44404* (*lincRNA-IBIN*). p-value <0.005 in all selected genes (S1 Table). **B)** 16 upregulated lncRNA genes have more than a threefold expression change in response to a *M*. *luteus* infection in adult flies. p-value <0.05 in all selected lncRNA genes (S2 Table). **C)** *lincRNA-IBIN* expression is induced in *Drosophila* adults within two hours of an infection by *M*. *luteus* or *E*. *cloacae*. For fold-induction values, expression values in uninfected samples were set to 1. **D)** *M*. *luteus*-induced *lincRNA-IBIN* expression is dependent on the Toll pathway (the Toll pathway adaptor protein MyD88) function. **E)** *E*. *cloacae-*induced *lincRNA-IBIN* expression is dependent on the Imd pathway (Relish) function. In **D** and **E**, for fold-induction values, expression values in uninfected *w*, *MyD88*^*IR*^ samples were set to 1 **F)** *lincRNA-IBIN* expression is induced in *Drosophila* adults upon silencing of the *Drosophila* inhibitor of κB factor *cactus*. **G)** *lincRNA-IBIN* expression is induced in *Drosophila* larvae with the constitutively active form of the Toll receptor, *Toll*^*10b*^. In **F** and **G**, for fold-induction values, expression values in uninfected/untreated *w* samples were set to 1. **H)** *lincRNA-IBIN* expression is also modestly induced by the ubiquitous overexpression of *Imd* with daughterless-GAL4 (*da>Imd*) in *Drosophila* larvae. For fold-induction values, the expression value of *w*,*da*> was set to 1. **I-J)** Both *M*. *luteus* and *E*. *cloacae*-induced *lincRNA-IBIN* expression is dependent on the functional chromatin remodeling BAP complex. In **I** and **J**, for fold-induction values, expression values in uninfected *w*, *osa*^*IR*^ samples were set to 1.

To investigate the importance of long non-coding RNAs in *Drosophila* immunity, we carried out a transcriptome analysis (RNAseq) of flies 24h after infection with the Gram-positive *M*.*luteus* in comparison to age and sex-matched uninfected controls. The RNA sequencing method used in this study recognizes polyadenylated non-coding RNAs, which are thought to represent the majority of long non-coding RNAs, although also ones without poly-A tails exist [25,26]. Prior to the transcriptome analysis, one of the Toll pathway target genes *IM1* (*Immune induced molecule 1*), was measured from females and males upon *M*. *luteus* infection. *IM1* was robustly induced in both male and female *Drosophila* (S1A Fig), and males were chosen for the transcriptome analysis. LYS-type peptidoglycan containing Gram-positive bacteria are known to induce the classical Toll pathway target genes including a number of antimicrobial peptides (AMPs) (e.g. [27]). As expected, AMPs were strongly upregulated in the transcriptome analysis upon a *M*. *luteus* infection, including *Dro*, *Mtk*, *Drs*, and multiple *IMs* (Fig 1A, S1 Table). Noteworthy, the highest upregulation in infected flies was seen in a previously unannotated long non-coding RNA gene, *CR44404* (Fig 1A). The baseline expression of *CR44404* is very low, and upon a *M*. *luteus* infection, it is induced by about 1300-fold. The induction of *CR44404* expression was also shown to be comparable between males and females upon *M*. *luteus* infection (S1B Fig).

Besides *CR44404*, there were only 15 other lncRNAs that were more than 3-fold upregulated upon infection (Fig 1B, S2 Table). While findings from vertebrates indicate that lncRNAs have wide and important functions in immune responses [13–16], cancer and metabolism [28,29], the role of lncRNAs in *Drosophila* immunity has only begun to emerge. *CR44404* was chosen for further analysis based on its intriguing expression pattern.

*CR44404* is 228 nucleotides long (genomic loci 2R:17,671,068..17,671,295 [+]) and it is located between two protein coding genes; *P32* and *CG30109*. Therefore, *CR44404* is classified as a long non-coding intergenic RNA (lincRNA) molecule. Although *CR44404* is very close to the protein-coding gene P32, the genes do not overlap. To confirm that *CR44404* is an independent transcript, the expression levels of the adjacent genes were examined in the transcriptome analysis. Neither *P32* nor *CG30109* were affected by infection in the same way as *CR44404*, the expression of which was ~1300-fold upon a *M*. *luteus* infection. Instead, *P32* (1.17-fold) and *CG30109* (1.27-fold) were not significantly induced by *M*. *luteus* infection at 24h time point, indicating that *CR44404* is expressed independently from them.

*CR44404* is polyadenylated; it has a highly conserved cleavage signal sequence *AAUAA* towards the end of the full-length transcript. *CR44404* does not contain open reading frames and based on the NCBI domain search tool [30], it does not contain any predicted protein domains. According to RNA secondary structure predictions, *CR44404* is multibranched (contains 3–4 GC-rich branches) and contains a variable amount of smaller (hairpin) loops connected to a bigger loop (S2 Fig). Based on the high expression of *CR44404* upon infection and its genomic location, we named the gene *lincRNA-Induced By INfection* (*lincRNA-IBIN*).

### *lincRNA-IBIN* expression is induced by Gram-positive and -negative bacteria and parasitoid wasps and is dependent on the functional Toll and Imd pathways and the BAP complex

As *lincRNA-IBIN* was shown to be strongly induced by Gram-positive bacteria 24h p.i, we next infected male flies with either the Gram-positive *M*. *luteus* or the Gram-negative *Enterobacter cloacae* (E. cloacae) to measure the gene expression kinetics of *lincRNA-IBIN* during multiple time points ranging from 0-24h after infection (Fig 1C). This revealed that *lincRNA-IBIN* is also induced by Gram-negative bacteria, and in both infections, the induction occurred within the first hours of infection and gradually increased towards the 24h time point (Fig 1C). To further study the role the Toll pathway and the Imd pathway [10,11], in the expression of *lincRNA-IBIN*, we knocked down *MyD88* (an adaptor protein functioning downstream of the Toll receptor in the Toll pathway), *cactus* (a negative regulator of the Toll pathway) and *Relish* (an Imd pathway NF-κB factor). Thereafter, we infected the flies with *M*. *luteus* or *E*. *cloacae* and measured the *lincRNA-IBIN* RNA levels. Upon a *M*. *luteus* infection, the expression of *lincRNA-IBIN* was shown to be dependent on the expression of *MyD88*, i.e the functional Toll pathway (Fig 1D). Knocking down *MyD88* upon a *E*. *cloacae* infection had no effect on the expression of *lincRNA-IBIN* (Fig 1E), whereas knocking down *Relish* or using the *Relish*^*E20*^ null mutant inhibited the expression of *lincRNA-IBIN*, showing that it requires a functional Imd pathway in this context (Fig 1E). Knocking down *Relish* or using the *Relish*^*E20*^ null mutant upon a *M*. *luteus* infection did not inhibit the expression of *lincRNA-IBIN* (Fig 1D). The role of the Toll pathway activation to the expression of *lincRNA-IBIN* was further confirmed in uninfected flies by knocking down the inhibitor of the κB factor *cactus*, which strongly induced the expression of *lincRNA-IBIN* (Fig 1F). Also, during the larval stage, *lincRNA-IBIN* was induced by the ectopic expression of the constitutively active form of the Toll receptor, *Toll*^*10b*^ (Fig 1G) and by overexpression of the Imd molecule with the ubiquitous *da-GAL4* driver (Fig 1H).

**Fig 2:**
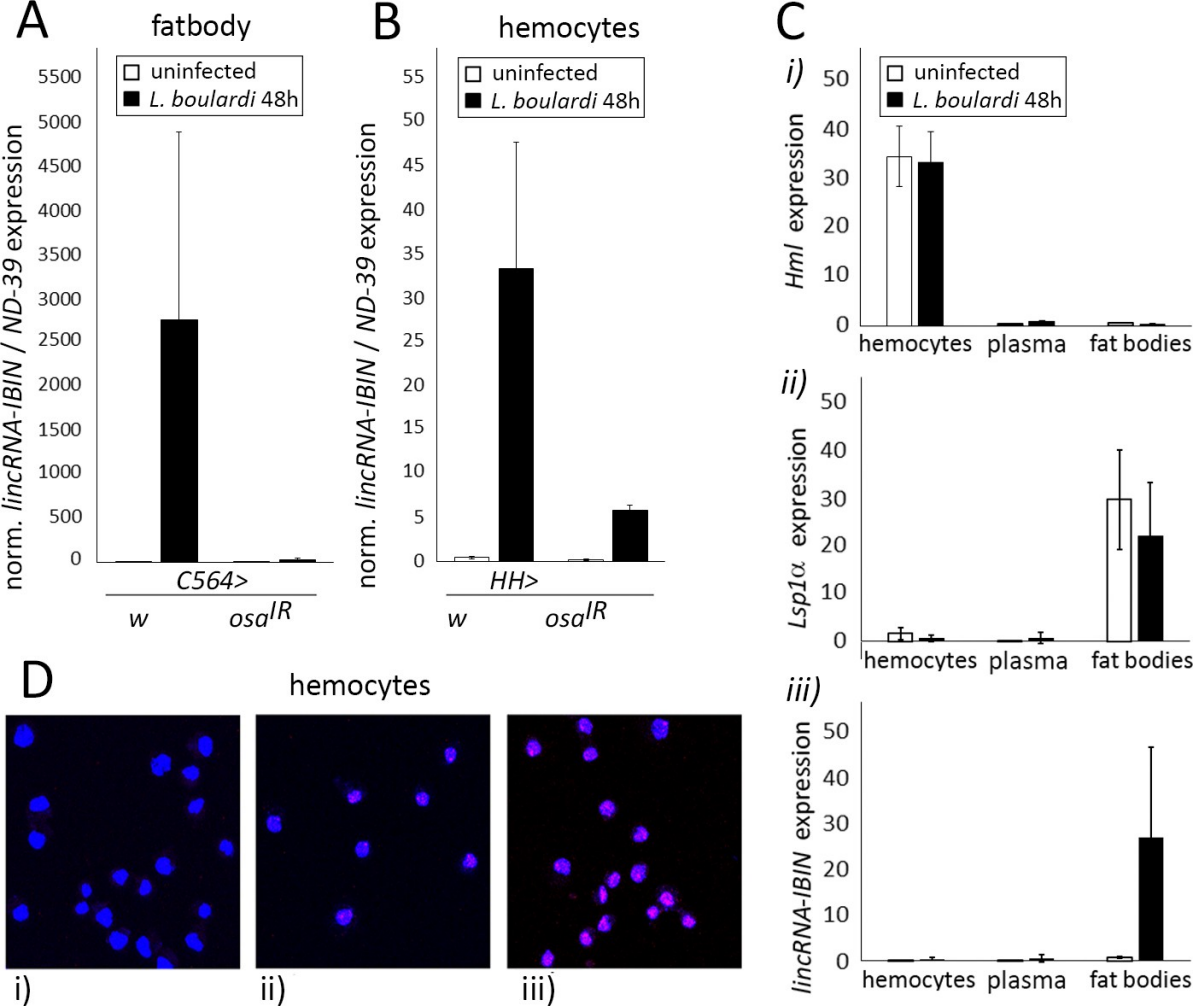
*lincRNA-IBIN* expression is induced in immunogenic tissues and its cellular localization is mainly nuclear. **A-B)** *lincRNA-IBIN* is induced in the larval fat body and hemocytes after a *L*. *boulardi* infection and is dependent on the expression of the BAP complex member *osa* in these tissues. For fold-induction values, expression values in uninfected *w*, *osa*^*IR*^ samples were set to 1. **C)** qPCR for hemocyte-specific *Hml* (i) and fat body-specific *Lsp1α* (ii) was carried out to confirm the purity of the tissue fractions. *lincRNA-IBIN* was not found in the plasma fraction in large quantities (iii). **D)** RNA FISH performed in larval hemocytes shows that *lincRNA-IBIN* is mainly located in the nucleus; pink labelling (*lincRNA-IBIN*) co-localizes with blue nuclear labelling (DAPI). **i)** Negative control (without *lincRNA-IBIN* probes), **ii)** hemocytes from *w*^*1118*^ larvae showing the basal expression level and localization of *lincRNA-IBIN*, **iii)** hemocytes from larvae overexpressing *lincRNA-IBIN*^*1*^ (*HH>lincRNA-IBIN*^*1*^) and infected with *L*. *boulardi*.

Next, we tested if the Osa-containing Brahma (BAP) complex is needed for the expression of the *lincRNA-IBIN*. The BAP complex is a group of protein-coding genes working together in remodeling chromatin [31], and the complex has previously been reported to affect the Toll pathway-induced *Drs-luc* reporter *in vitro* in *Drosophila* [32,33]. Interestingly, when *osa* expression was knocked down, *lincRNA-IBIN* expression was strongly inhibited upon both a *M*. *luteus* (Fig 1I) and a *E*. *cloacae* (Fig 1J) infection. The knockdown of another BAP complex component *brahma* (*brm*) also reduced *lincRNA-IBIN* expression upon both infections (Fig 1I and 1J). Moreover, because *lincRNA-IBIN* is strongly induced by a bacterial infection in *Drosophila* adults, indicating a role in the humoral immune response, we next studied whether *lincRNA-IBIN* is also induced during the cellular immune response by infecting *Drosophila* larvae with *Leptopilina boulardi* (L. boulardi) parasitoid wasps. Also in this context, the expression of *lincRNA-IBIN* was strongly induced (Fig 2).

In conclusion, *lincRNA-IBIN* seems to have a rather broad role in the immune response, being induced by a bacterial infection in flies and by parasitoid wasps in larvae. The *M*. *luteus* -mediated induction of *lincRNA-IBIN* expression was shown to be dependent on the Toll pathway, whereas the *E*. *cloacae* -mediated induction requires the Relish/Imd pathway. In each studied case, *lincRNA-IBIN* expression was dependent on a functional BAP complex. This type of unspecific induction via both NF-κB pathways is rather uncommon in *Drosophila* and argues for a general immunity related function for *lincRNA-IBIN*.

### Tissue-specific expression and cellular localization of *lincRNA-IBIN*

To understand the role of *lincRNA-IBIN* in *Drosophila* immunity, we investigated where *lincRNA-IBIN* was expressed and whether its effects were tissue-specific. Since *lincRNA-IBIN* expression is strongly infection-inducible, we reasoned that it is most likely expressed in immune-responsive tissues (the fat body and hemocytes, the *Drosophila* blood cells). Wasp infection of *Drosophila* larvae led to the induction of *lincRNA-IBIN* expression in the fat body and hemocytes (Fig 2A and 2B). Like in flies, *osa* RNAi in larval hemocytes (*HH> osa*^*IR*^) and fat bodies (*C564> osa*^*IR*^) kept the expression of *lincRNA-IBIN* close to the basal level (Fig 2A and 2B). Because *lincRNA-IBIN* is a short gene and very strongly induced upon infection like AMPs, we next investigated whether *lincRNA-IBIN* is secreted into the plasma in similar manner as AMPs (Fig 2C). First, we confirmed that hemocytes and plasma were separated by centrifugation (S3 Fig). We also checked the expression of a hemocyte specific gene *Hemolectin* (*Hml*) and a fat body-specific gene *Larval serum protein 1 alpha* (*Lsp1α*) in each tissue sample (Fig 2C, i and ii). A *Hml* signal was detected in the hemocyte fraction, whereas *Lsp1α* levels were high in fat body samples but not in hemocytes or in the plasma (Fig 2C, i and ii). We did not detect *lincRNA-IBIN* in the plasma fraction in large quantities (Fig 2C, iii).

Pin-pointing the cellular localization of a *lncRNA* reveals typically more about its function than does the structure of the RNA. A RNA FISH (*RNA Fluorescent In Situ Hybridization*) protocol was performed with 3rd instar larval hemocytes. Uninfected *w*^*1118*^ larval hemocytes were used as a control for imaging the basal expression level and localization of *lincRNA-IBIN* (Fig 2D, ii). Hemocytes from larvae overexpressing *lincRNA-IBIN*^*1*^ (*HH>lincRNA-IBIN*^*1*^) and infected with *L*. *boulardi* were used to induce the expression of *lincRNA-IBIN* (Fig 2D, iii), and they showed that *lincRNA-IBIN* (pink labelling) was primarily expressed in the nuclear compartment (blue labelling) of the cell. Therefore, we conclude that *lincRNA-IBIN* is expressed in immune responsive tissues, is not secreted into the plasma in large amounts and its cellular localization is mainly nuclear. This suggests that the function of *lincRNA-IBIN* may be in the regulation of gene expression, which is typical for lncRNAs [34–36].

### Overexpressing *lincRNA-IBIN* enhances the expression of selected AMPs upon an infection and survival from an infection

**Fig 3:**
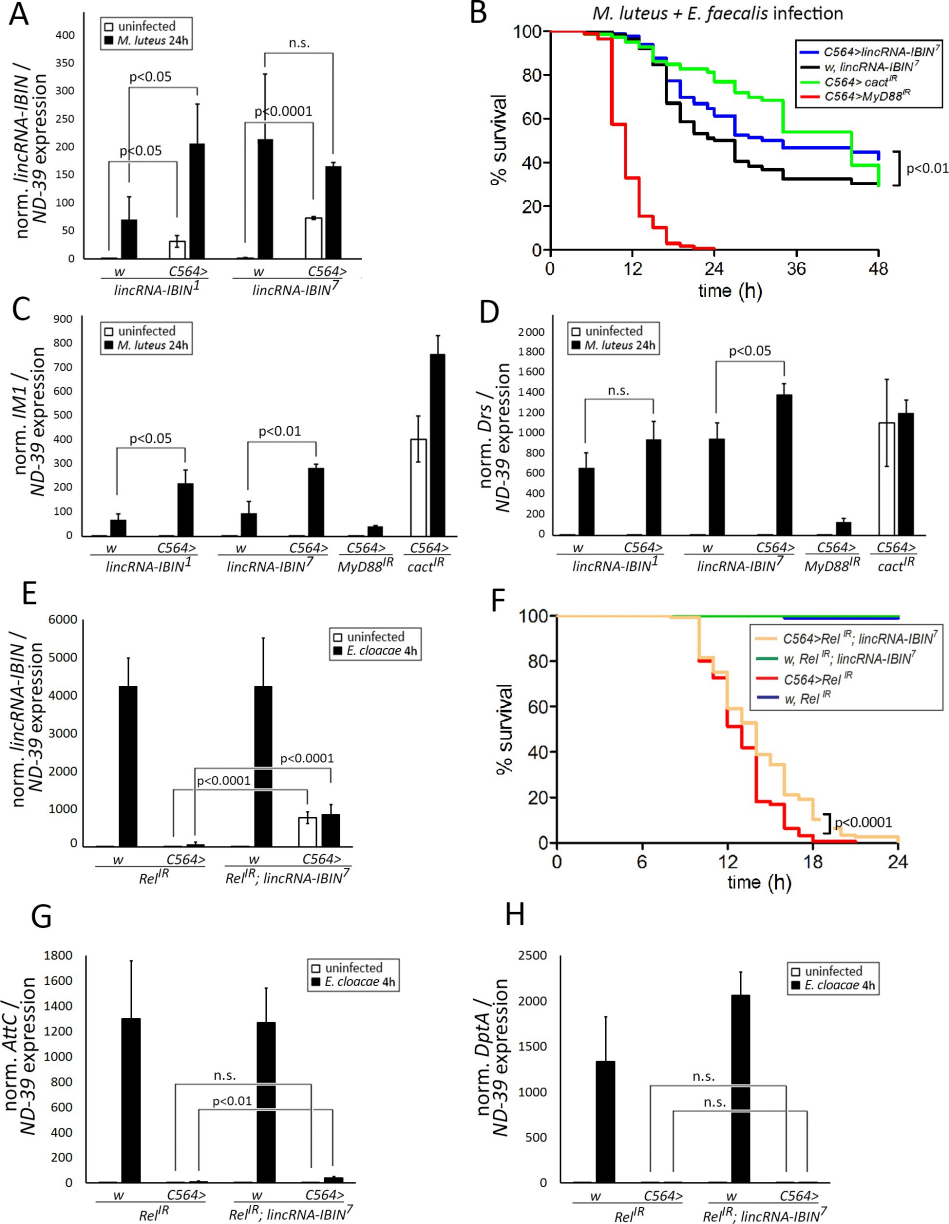
Overexpressing *lincRNA-IBIN* improves the survival of *Drosophila* adults from an infection and the expression of selected target genes. **A)** *UAS-lincRNA-IBIN* (*CR44404*) overexpression with the *C564-GAL4* driver (*C564>lincRNA-IBIN*^*1*^ and *C564>lincRNA-IBIN*^*7*^) significantly increases the expression of *lincRNA-IBIN* in uninfected flies measured with qPCR, as does an infection with *M*. *luteus*. **B)** *lincRNA-IBIN* overexpression (*C564>lincRNA-IBIN*^*7*^) improves the survival of the flies after an infection with *M*. *luteus* + *E*. *faecalis*. **C-D)** *lincRNA-IBIN* overexpression increases the expression of two Toll pathway target genes *IM1* **(C)** *and Drosomycin* **(D)** after a *M*. *luteus* infection. *MyD88* knockdown flies were used as a negative control and *cactus* knockdown flies as a positive control. In **A, C** and **D**, for fold-induction values, expression values in uninfected *w*, *lincRNA-IBIN*^*7*^ samples were set to 1. **E)** *E*. *cloacae*-induced endogenous *lincRNA-IBIN* expression is lost upon *Relish* RNAi. *lincRNA-IBIN* overexpression is shown in the Relish RNAi background. **F)** Upon a *E*. *cloacae* infection, *lincRNA-IBIN* overexpression gives a statistically significant survival advantage to flies with a *Relish* RNAi background. **G)** *Attacin C* (*AttC*) is not produced in *Relish* RNAi flies, but with IBIN overexpression a very small amount of *AttC* is induced. H) *Diptericin A* (*DptA*) is not produced with or without *lincRNA-IBIN* overexpression in flies with a *Relish* RNAi background upon a *E*. *cloacae* infection. In **E, G** and **H**, for fold-induction values, expression values in uninfected *w*, *Rel*^*IR*^ samples were set to 1.

To study the function of *lincRNA-IBIN* in uninfected and infected flies, we generated *UAS-lincRNA-IBIN* overexpression fly lines. Two of the generated lines, *lincRNA-IBIN*^*1*^ and *lincRNA-IBIN*^*7*^, were selected for the following experiments. *lincRNA-IBIN* overexpression in the *lincRNA-IBIN*^*1*^ and *lincRNA-IBIN*^*7*^ lines was induced using the *C564-GAL4* driver, which is expressed strongly in the fat body [37,38](Fig 3A). *lincRNA-IBIN* expression in uninfected flies was significantly increased in both overexpression lines (Fig 3A, white bars), with higher expression levels in the *lincRNA-IBIN*^*7*^ line. 24 h after a *M*. *luteus* infection, the effect of the overexpression on the expression of *lincRNA-IBIN* was masked by the overwhelming endogenous expression of *lincRNA-IBIN* (Fig 3A, black bars). To study the effect of the long-term exposure of flies to elevated levels of *lincRNA-IBIN*, we monitored the lifespan of flies overexpressing *lincRNA-IBIN* with the *C564-GAL4* driver and controls. To ensure maximal *lincRNA-IBIN* expression, flies were cultured at +29°C for the duration of the experiment. Neither one of the *lincRNA-IBIN* overexpression lines (*lincRNA-IBIN*^*1*^ and *lincRNA-IBIN*^*7*^) showed a statistically significant difference in the lifespan between flies overexpressing *lincRNA-IBIN* and controls (S4 Fig).

As *lincRNA-IBIN* was the most strongly induced gene upon a *M*. *luteus* infection, we first investigated whether overexpressing *lincRNA-IBIN* affected the survival of the flies against a septic infection with Gram-positive bacteria. For the survival experiment, we chose *lincRNA-IBIN*^*7*^ flies as these produced the highest overexpression without an infection. We first infected *lincRNA-IBIN*^*7*^ flies with *M*. *luteus* to prime the Toll pathway. 24h later, the flies were infected with the more pathogenic bacteria, *Enterococcus faecalis* (*E*. *faecalis*) [39]. Overexpression of *lincRNA-IBIN*^*7*^ (*C564-GAL4>lincRNA-IBIN*^*7*^) improved the survival of the flies from the infection compared to the flies not overexpressing *lincRNA-IBIN* (Fig 3B). *MyD88* (a positive regulator of the Toll pathway) and *cactus* (a negative regulator of the Toll pathway) knock-down flies were used as controls. This indicates that *lincRNA-IBIN* positively affects immunity against the pathogenic Gram-positive bacteria *E*. *faecalis*.

Next, we investigated whether *lincRNA-IBIN* regulates the central feature of the fly immune defense against Gram-positive bacteria, namely the production of AMPs via the Toll pathway. The expression of the Toll pathway mediated genes was monitored in flies overexpressing *lincRNA-IBIN* and controls after exposure to *M*. *luteus* for 24 hours (Fig 3C and 3D). *lincRNA-IBIN* overexpression using both the *lincRNA-IBIN*^*1*^ and *lincRNA-IBIN*^*7*^ lines with the *C564-GAL4* driver resulted in significantly elevated levels of *IM1* upon infection (Fig 3C). The expression of *Drosomycin* was elevated in *C564>lincRNA-IBIN*^*7*^ flies, whereas in the *lincRNA-IBIN*^*1*^ line the trend was similar, yet not significant (Fig 3D). As expected, *MyD88* knockdown decreased the expression of *IM1* and *Drosomycin* upon infection, whereas *cactus* knockdown caused a strong induction of *IM1* and *Drosomycin* expression also in the uninfected flies (Fig 3C and 3D).

To address the importance of *lincRNA-IBIN* in a situation where the expression of endogenous *lincRNA-IBIN* is prevented, we utilized the following experimental approach. Upon an *E*. *cloacae* infection, *lincRNA-IBIN* expression is fully dependent on Relish (Fig 1E). *Relish* RNAi flies do not produce endogenous *lincRNA-IBIN* upon an *E*. *cloacae* infection, but in the *Relish* RNAi flies combined with the *lincRNA-IBIN*^*7*^ construct, *lincRNA-IBIN* is overexpressed (Fig 3E). Next, we monitored the survival of *Relish* RNAi flies and *Relish* RNAi flies with the *lincRNA-IBIN*^*7*^ construct from an *E*. *cloacae* infection (Fig 3F). Fig 3F demonstrates that *lincRNA-IBIN* overexpression provides protection against an *E*. *cloacae* infection. *lincRNA-IBIN* overexpression does not itself induce antimicrobial peptides (Fig 3G and 3H), indicating that the protection is independent of the AMPs. Taken together, *lincRNA-IBIN* overexpression enhances the expression of target genes of the Toll pathway. Upon infection, *lincRNA-IBIN* overexpression gave flies a survival advantage. However, this is not due to the induction of AMPs itself, but results from a mechanism that prompts further investigation.

### Overexpression of *lincRNA-IBIN* in hemocytes increases hemocyte numbers

As shown in Fig 2, *lincRNA-IBIN* is expressed in immunogenic tissues in the fly, such as the fat body and hemocytes. Next, we examined the role of *lincRNA-IBIN* overexpression in the cellular response, i.e. the hemocytes. Phagocytic plasmatocytes are the main hemocyte type in uninfected larvae. Lamellocytes, which are formed upon a parasitoid wasp infection, function in the encapsulation of the wasp eggs and larvae [5,40,41]. To further investigate if *lincRNA-IBIN* has a role in two major components of the cellular immune response, namely the increase in hemocyte numbers and differentiation of lamellocytes, we utilized the hemocyte reporters (*msnCherry*,*eaterGFP*) to detect hemocytes with flow cytometer. The combination of the reporters with the hemocyte (*MeHH>* for short, see materials and methods) and fat body (*MeC564>*) drivers enabled us to detect the hemocytes and overexpress *lincRNA-IBIN* in these tissues. Driving *lincRNA-IBIN* expression in hemocytes (*MeHH>lincRNA-IBIN*) resulted in an increase in total hemocyte numbers in uninfected larvae (S5A Fig), but did not induce ectopic lamellocyte formation (S5A’ Fig). *lincRNA-IBIN* overexpression in the fat body (*MeC564>lincRNA-IBIN*) did not have an effect on hemocytes (S5B–S5B’ Fig).

*lincRNA-IBIN* could enhance the proliferation of hemocytes or their release from a reservoir located in segmental bands under the larval cuticle, called the sessile compartment [42,43]. To that end, we imaged whole larvae and checked for the existence of sessile bands. We did not observe any noticeable loss of sessile bands that could explain the increased hemocyte numbers (S5C Fig). In *L*. *boulardi*-infected larvae, there was a slight decrease in the numbers of hemocytes in the *lincRNA-IBIN*^*7*^ line (S5A Fig), but lamellocytes were not affected (S5A’ Fig). Taken together, the overexpression of *lincRNA-IBIN* in hemocytes increases the hemocyte numbers, but does not affect hemocyte differentiation, in unchallenged *Drosophila* larvae.

### Transcriptome profiling of flies overexpressing *lincRNA-IBIN* shows effects on sugar and amino acid metabolism

**Fig 4:**
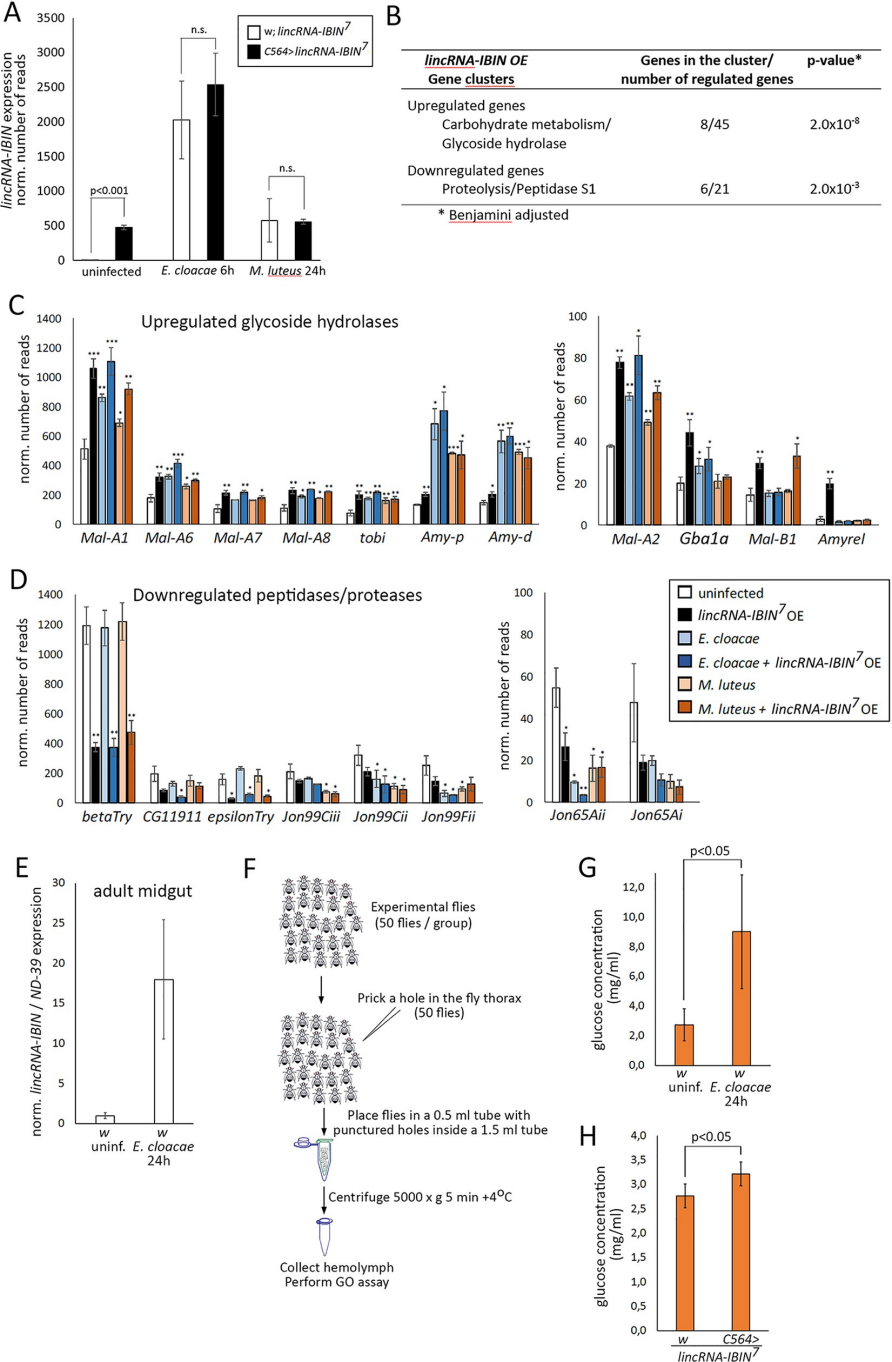
Transcriptome analysis of *lincRNA-IBIN* overexpressing flies reveals changes in metabolic genes that result in increased glucose levels in the hemolymph. **A)** *lincRNA-IBIN*^*7*^ overexpression with the *C564-GAL4* driver increases *lincRNA-IBIN* expression in uninfected flies by 161-fold. This induction is not visible upon a *E*. *cloacae* or *M*. *luteus* infection, since the infection induces strong *lincRNA-IBIN* expression. **B)** Cluster analysis of genes up-and downregulated in *C564>lincRNA-IBIN* flies compared to *w; lincRNA-IBIN* controls. The major cluster of induced genes consists of glycoside hydrolase enzymes, whereas enzymes involved in proteolysis form the main downregulated cluster. Clustering was done using GO Biological Process and InterPro protein classification (GO BP/InterPro). **C,D)** Expression differences of selected *Drosophila* genes involved in metabolism with and without *lincRNA-IBIN* overexpression and with and without an infection based on the transcriptome analysis. **E)** *lincRNA-IBIN* is induced in the *Drosophila* adult midgut by a *E*. *cloacae* septic injury 24h post infection. **F)** Workflow of the collection of hemolymph for glucose and trehalose measurements. **G,H)** Circulating hemolymph glucose levels are elevated upon **G)** infection and **H)** overexpression of *lincRNA-IBIN* in the adult midgut.

To identify the downstream pathways and targets of *lincRNA-IBIN*, we performed transcriptome profiling of flies overexpressing (OE) the *lincRNA-IBIN*^*7*^ construct with the *C564-GAL4* driver. *lincRNA-IBIN* OE and control flies were either infected with *M*. *luteus* or *E*. *cloacae*, or they were left uninfected. In uninfected *lincRNA-IBIN* OE flies, *lincRNA-IBIN* expression was induced 166-fold (Fig 4A (472±33 normalized number of reads for *C564>IBIN* vs. 2.8±0.62 normalized number of reads for *w*, *IBIN*). Also in this transcriptome analysis, target genes of the Toll pathway were induced in *M*. *luteus*-infected flies, and *lincRNA-IBIN* OE further elevated their levels (S3 Table). Candidate target genes under *lincRNA-IBIN* regulation were searched for in the uninfected *lincRNA-IBIN*^*7*^ transcriptome data using a cut-off value of 2 for fold change. Genes with induced expression level from medium to high (>10 reads) in the treatment of interest were included in the analysis. Based on this criteria, 45 genes (including *lincRNA-IBIN*) were upregulated (S4 Table) and 21 genes were downregulated (S5 Table) in unchallenged *lincRNA-IBIN* OE flies. The top upregulated genes included one of the copies of *Major heat shock 70 kDa protein*, *Hsp70Bb* (32.9-fold), *Niemann-Pick type C-2* (*Npc2e*, 29.6-fold) and *Amyrel* (6.8-fold; S4 Table). Among the most downregulated genes were *εTrypsin* (5.3-fold) and *βTrypsin* (3.2-fold) both involved in proteolysis (S5 Table). A cluster analysis of the up- and downregulated genes revealed two major gene clusters, both of which are involved in metabolism (Fig 4B). Eight out of forty-five genes upregulated in *lincRNA-IBIN* overexpressing flies belong to a carbohydrate metabolism/glycoside hydrolase gene cluster, and six out of twenty-one downregulated genes belong to a proteolysis / peptidase S1 gene cluster (Fig 4B).

To investigate the identified metabolism related gene clusters in more detail, we plotted normalized read values of selected genes including all the treatments (uninfected, *lincRNA-IBIN*^*7*^ OE, *E*. *cloacae* and *M*.*luteus* infections with and without *lincRNA-IBIN*^*7*^ OE; Fig 4C & 4D). As shown in Fig 4C, overexpression of *lincRNA-IBIN*^*7*^ in uninfected flies causes the upregulation of six maltase genes (*Mal-A1*, *Mal-A6*, *Mal-A7*, *Mal-A8*, *Mal-A2*, *Mal-B1*), whose function is to catalyze the hydrolysis of maltose (disaccharide) to glucose units (monosaccharide). In addition, *lincRNA-IBIN*^*7*^ overexpression increased the expression of amylases (*Amy-p*, *Amy-d*, *Amyrel*), which hydrolyze dietary starch into disaccharides. *tobi* (*target of brain insulin*) and *Gba1a* (*Glucocerebrosidase 1a*) are also involved in sugar metabolism, and were elevated by *lincRNA-IBIN*^*7*^ overexpression (Fig 4C). Of note, the expression of some of these genes involved in carbohydrate metabolism was also elevated upon *M*. *luteus* and *E*. *cloacae* infections (Fig 4C, blue and orange bars). This demonstrates that enhanced sugar metabolism is needed to fight infections as shown before [44–46], and indicates that *lincRNA-IBIN* is involved in providing this metabolic switch. In contrast, many genes involved in peptide and protein catabolism were downregulated upon *lincRNA-IBIN*^*7*^ overexpression and infection (Fig 4D). The enhanced (S6B and S6C Fig) or downregulated (S6D Fig) expression of selected metabolic genes upon *lincRNA-IBIN* overexpression (S6A Fig) was confirmed by qPCR also with the other *lincRNA-IBIN* overexpressing fly line, *lincRNA-IBIN*^*1*^.

### *lincRNA-IBIN* is expressed in the adult midgut upon a septic infection and modulates glucose levels in the hemolymph

According to the Flybase high throughput expression data (www.flybase.org), all the identified metabolism genes are almost exclusively expressed in the midgut. Therefore we next analyzed whether *lincRNA-IBIN* is expressed in the adult midgut in response to septic injury. To ensure maximal induction of *lincRNA-IBIN*, we infected adult flies by pricking them with *E*. *cloacae*, and 24h later dissected the midgut tissues of the infected flies and controls. An *E*. *cloacae* infection induced the expression of *lincRNA-IBIN* also in the adult midgut (Fig 4E). Furthermore, strong expression of the *C564-GAL4* driver in the adult midgut was demonstrated (S7A Fig), indicating that besides the fat body, *lincRNA-IBIN* is also overexpressed in the midgut when the driver *C564-GAL4* is used (S7B Fig). This is in line with the observed transcriptional changes when the overexpression of *lincRNA-IBIN* was driven using the *C564-GAL4* driver.

To further study changes in glucose metabolism during an infection, we measured glucose and trehalose levels from the hemolymph 24h after a septic injury with *E*. *cloacae*. Hemolymph from uninfected and infected flies (50 flies in each sample) was collected for the analysis (Fig 4F). An infection induced an increase in the glucose level in circulating hemolymph (Fig 4G), whereas the level of circulating trehalose was unaltered (S7C Fig). This suggests that increasing the hemolymph glucose level is important for the immune response. Next, to investigate the role of *lincRNA-IBIN* on the hemolymph glucose level, hemolymph from flies overexpressing *lincRNA-IBIN*^*7*^ with the *C564-GAL4* driver and controls (*w*; *lincRNA-IBIN*^*7*^) was collected as described (Fig 4F). In addition to an infection (Fig 4G), also *lincRNA-IBIN*^*7*^ overexpression caused a statistically significant increase in the glucose level in circulating hemolymph (Fig 4H). In conclusion, these data imply that a septic infection with Gram-negative bacteria enhances the transcription of genes involved in carbohydrate metabolism, which leads to elevated sugar levels in the hemolymph. *lincRNA-IBIN* regulates in part this metabolic shift to ensure sufficient energy resources for the needs of the immune cells and tissues.

## Discussion

*Drosophila melanogaster* has been one of the most fruitful models for studying the immune response [1]. For example, identification of the regulators of the signaling cascades of innate immunity in *Drosophila* has greatly advanced our understanding of the control of mammalian immune responses [3,47]. Today, key pathways and proteins controlling the immune reactions in *Drosophila* are well documented [10,11,48,49]. However, while findings in vertebrates indicate that lncRNAs have wide and important functions in immune responses [13–16], cancer and metabolism [26, 27], the role of lncRNAs in *Drosophila* immunity has only begun to emerge.

Based on our *Drosophila* transcriptome analysis, only few lncRNA genes were up- or downregulated in response to an infection with the Gram-positive LYS-type peptidoglycan containing bacteria *M*. *luteus*. However, expression of the gene *CR44404*, named as *lincRNA-IBIN* (*I**nduced* *B**y* *IN**fection*), was highly upregulated during a *M*. *luteus* infection in flies. *lincRNA-IBIN* expression was also induced by the Gram-negative DAP-type peptidoglycan containing bacteria *E*. *cloacae*, indicating that activation of either the Toll or the Imd pathway induces its expression. As an infection with the parasitoid wasp *L*. *boulardi* also induced the expression of *lincRNA-IBIN*, this indicates that *lincRNA-IBIN* might have a general role in immunity.

The expression patterns of lncRNAs have been found to be highly specific for tissue, developmental stage and context (reviewed in [14,15]). When studying the expression levels of *lincRNA-IBIN* in larvae, the highest expression levels were found in the fat body. *lincRNA-IBIN* was also expressed in hemocytes, but was not secreted in considerable amounts into the plasma. The basal level of *lincRNA-IBIN* expression in these tissues was very low. However, the *lincRNA-IBIN* response to an infection was fast, as already at two hours after a bacterial infection the expression of *lincRNA-IBIN* was highly induced. This shows similar expression kinetics to AMPs and further argues for an important function for *lincRNA-IBIN* in immunity.

The functional importance of *lincRNA-IBIN* was studied by overexpressing it in the fat body and in hemocytes with the UAS-GAL4-system. Overexpressing *lincRNA-IBIN* locally in the fat body resulted in elevated levels of the antimicrobial peptide *Drosomycin* and another Toll pathway target, *IM1*, upon a *M*. *luteus* infection. *lincRNA-IBIN* overexpression also led to enhanced resistance against an infection with Gram-positive bacteria. To study the function of *lincRNA-IBIN* further, we performed a full transcriptome analysis of uninfected and infected flies overexpressing *lincRNA-IBIN*. The most upregulated gene in *lincRNA-IBIN* overexpressing flies was *Hsp70Bb*. The main function of the Hsp70 proteins, like other heat shock proteins, is to maintain the proper trafficking and folding of proteins [50]. In addition, *Hsp70* expression has been shown to be induced by the Gram-negative *Erwinia carotovora carotovora* [51] and by medium from γ-irradiated *Escherichia coli* bacteria [52]. As a stress-responsive gene, the upregulation of *Hsp70Bb* could indicate that *lincRNA-IBIN* overexpressing flies are experiencing stress. However, no other heat shock genes were induced in *lincRNA-IBIN* OE flies, indicating that a general stress response is unlikely. Another highly upregulated gene in *lincRNA-IBIN* overexpressing flies was *Npc2e*. Npc2e binds microbial components, and it has been indicated to play a role in the Imd pathway [53]. In our data, *Npc2e* was also upregulated after *E*. *cloacae* and *M*. *luteus* infections (S4 Table), but these changes were not significant after an FDR correction.

Importantly, the *lincRNA-IBIN* OE transcriptome analysis revealed two main gene clusters, which were affected by *lincRNA-IBIN* overexpression, namely glycoside hydrolases (upregulation) and peptidases and proteases (downregulation). Inflammatory responses from infection to cancer involve changes in metabolic pathways [54,55]. The activated immune cells switch from oxidative phosphorylation toward aerobic glycolysis; this is a well-known phenomenon in cancer cells called the Warburg effect, and it is also recognized as important for immune cells upon an infection [44,56,57]. The metabolic switch toward aerobic glycolysis leads to increased glucose consumption [58]. In *Drosophila*, the polysaccharide starch can be hydrolyzed into the disaccharide maltose and further on into the monosaccharide glucose by maltases. *lincRNA-IBIN* overexpression elevated the expression of multiple maltase genes, indicating that *lincRNA-IBIN* has a role in enhancing the catabolism of starch. Here we showed that besides the fat body cells and hemocytes, *lincRNA-IBIN* was also expressed in the *Drosophila* gut upon infection, which is the main site of starch metabolism. *lincRNA-IBIN* overexpression with the *C564-GAL4* driver, which is also strongly induced in the midgut, led to elevated levels of free glucose in adult hemolymph providing an energy source for immune cells. *lincRNA-IBIN* did not seem to affect the insulin signalling pathway, which in humans, and in flies, controls peripheral as well as central nervous system -related aspects of metabolism [59,60].

Glucose and trehalose are the major circulating sugars in *Drosophila*, and the majority of the total sugar in the hemolymph is trehalose [61]. *lincRNA-IBIN* overexpression enhanced the expression of glycoside hydrolases and led to slightly elevated levels of hemolymph glucose, but not trehalose. Therefore, *lincRNA-IBIN* may improve the retrieval of glucose from dietary sugars from the gut, for example by enhancing the expression of maltases. More elevated glucose levels were seen by a septic infection. It is likely that upon infection, also other factors influence the elevation of glucose levels compared to *lincRNA-IBIN* overexpression alone; for example, the enhanced release of glucose from glycogen storages upon infection has been shown [44,46].

The expression of genes related to proteolysis was downregulated upon *lincRNA-IBIN* overexpression, indicating that the uptake of amino acids from food is reduced. Immune responses are tightly regulated as prolonged inflammation is costly to the host [62,63]. Dionne and coworkers showed that flies infected with *Mycobacterium marinum* undergo a process resembling wasting, where the flies progressively lose metabolic stores in the form of fat and glycogen and become hyperglycemic [45]. Hence, there must be delicately controlled mechanisms for the direct control of energy allocation to the immune response upon infection or inflammation. Here, we have characterized *lincRNA-IBIN* as one of the potential regulators of this switch.

It is important to notice, however, that we have used UAS/GAL4-based overexpression of *lincRNA-IBIN* to study its function. We trust that this artificial overexpression system provides valid information about the effect of *lincRNA-IBIN* on its target genes, but one cannot exclude that some of the observed effects may be caused by non-physiological *lincRNA-IBIN* expression. For example, overexpressing a short RNA molecule, such as *lincRNA-IBIN*, may trigger antiviral immune responses. However, the normal life span and the lack of elevated expression of known virus infection-responsive genes such as *Vago*, *AGO2* or *Sting* [64–66] in *lincRNA-IBIN* OE flies argues against a non-specific viral response. However, the more definite conclusion about the role of *lincRNA-IBIN* in *Drosophila* immunity will require the generation of a loss-of-function mutant. *lincRNA-IBIN* mutant flies would also be essential to address whether *lincRNA-IBIN* is required for the observed metabolic changes during an infection and to address if *lincRNA-IBIN* is required for normal innate immunity in *Drosophila*.

*lincRNA-IBIN*, like most lncRNAs, demonstrates low evolutionary sequence conservation, and the lack of homologous sequences prevented the use of similar sequences for the identification of a function for *lincRNA-IBIN*. lncRNAs are composed of domains that permit either protein binding and/or base-pairing with RNA or DNA sequences [34,67–70]. Based on a secondary structure prediction, it is difficult to estimate whether *lincRNA-IBIN* interacts with protein, DNA or RNA molecules. The primary functions of lncRNAs can be traced according to their cellular localization. Based on our *RNA fluorescent in situ hybridization* analysis, *lincRNA-IBIN* was mostly localized within the nucleus. The nuclear localization suggests that *lincRNA-IBIN* localizes to its genomic target site(s) through RNA-DNA or RNA-protein interactions in the chromatin, where it could modulate chromatin regions or the expression of target genes by functioning as a guide, decoy or a scaffold for interacting molecules.

**Fig 5:**
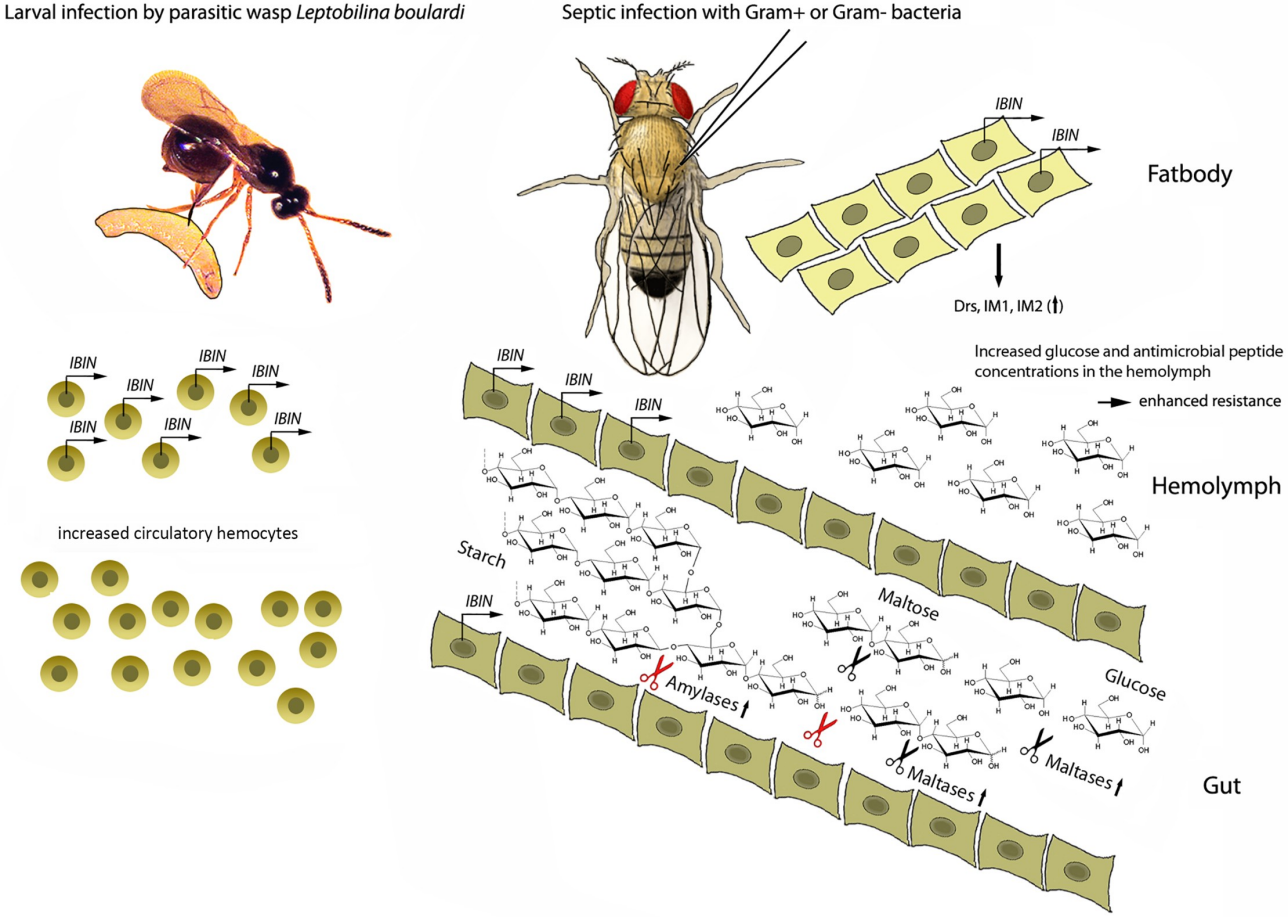
Schematic representation of *lincRNA-IBIN* functions in *Drosophila* larvae and adults.

An overview of *lincRNA-IBIN* functions is presented in Fig 5. Taken together, *lincRNA-IBIN* has a role in both humoral and cellular innate immune pathways in larvae and adults. The basal expression level of *lincRNA-IBIN* is very low and it responds rapidly to an infection, but further studies are required to fully understand its role in different infection contexts. *lincRNA-IBIN* is expressed in immune responsive tissues and its expression is regulated by NF-κB signaling and the chromatin modeling BAP complex. In the gut, *lincRNA-IBIN* has a role in the activation of glycoside hydrolases. Finally, expression of *lincRNA-IBIN* elevates the levels of free glucose in the hemolymph. Based on our findings we postulate that *lincRNA-IBIN* has an important role in the metabolic switch required to provide additional glucose for immune cells during a systemic infection in *Drosophila*.

## Materials and methods

### *Drosophila* lines and fly husbandry

The UAS-GAL4 -based system in *Drosophila* was utilized in most experiments to achieve the silencing or overexpression of genes in the F1 progeny [71]. The *brm* (transformant ID #37720), *osa* (#7810), *MyD88* (#25399) and Relish (#108469) UAS-RNAi lines, and the isogenized *w*^*1118*^ flies (*w*^*1118iso*^) that were used as controls, were obtained from the Vienna *Drosophila* Resource Center (www.vdrc.at). *cactus* UAS-RNAi flies (5848R-3) were obtained from the National Institute of Genetics Fly Stock Center in Japan. *Relish*^*E20*^ mutant flies were a kind gift from prof. Dan Hultmark. The *Imd* overexpression line that overexpresses the Imd protein under the control of UAS, was originally a kind gift from prof. Jules Hoffmann. The constitutively active *Toll*^*10b*^ mutant [72] was originally obtained from the Bloomington *Drosophila* Stock Center at Indiana University. *C564-GAL4* flies, driving the expression of a UAS-construct in the fat body [37,38,73] and some other tissues, were a kind gift from Prof. Bruno Lemaitre (Global Health Institute, EPFL, Switzerland). The *Daughterless-GAL4* (*Da-GAL4*) flies drive the expression of a UAS-construct ubiquitously [74]. The combination of the *Hml*^*Δ*^*-GAL4* (*w*^*1118*^*; P{Hml-GAL4*.*Δ}2P{wUAS-2xEGFP}AH2*) driver [75] and the *He-GAL4* (*P{He-GAL4*.*Z}*) driver [76](*HH-GAL4*) was used to drive the expression of the UAS-constructs in hemocytes [77]. The GAL4 drivers were backcrossed into the *w*^*1118*^ background that was used as a control in crosses without a GAL4-driver. The hemocyte reporter lines *eaterGFP* (for plasmatocytes) [78] and *MSNF9mo-mCherry* (for lamellocytes, hereafter called *msnCherry*) [79] were obtained from Robert Schulz’s laboratory. The lines were recombined to create the *msnCherry*,*eaterGFP* reporter line. The *msnCherry*,*eaterGFP* reporter was further crossed with *C564-GAL4* to obtain *msnCherry*,*eaterGFP;C564-GAL4* (*MeC564>* for short). *msnCherry*, *eaterGFP;* *Hml*^*Δ*^*-GAL4;* the *He-GAL4* (*MeHH>*) line was a kind gift from I. Anderl.

To create *lincRNA-IBIN* overexpressing fly lines, the full-length gene for *lincRNA-IBIN* was cloned into the *EcoRI* and *BcuI* (*SpeI*) restriction sites in the pUAST vector using the following primers (with restriction sites underlined): CR44404_F: TAAGCAGAATTCCACAATCTAAAGTTAACTTGCC and CR44404_R: CACACAACTAGTGTTTATTTTCTTTCTATGGTTG. The produced plasmids were injected into the *w*^*1118*^ background in Best Gene Inc., USA (thebestgene.com). Ten lines producing red-eyed transformants were generated, and two lines (lincRNA-IBIN^1^ and lincRNA-IBIN^7^) with good overexpression of *lincRNA-IBIN* were selected for experiments.

For the experiments, 10–15 virgin females were crossed with 5–7 males per vial containing mashed-potato, syrup and yeast-based fly food medium. Crosses were kept at +25°C and flies transferred daily into fresh vials. The vials with eggs were transferred to +29°C after one day of egg laying and kept there until the experiments at the larval or adult stage unless otherwise stated. Test groups and controls were kept at the same conditions at all times. After testing that target genes of the Toll pathway were induced in a similar manner in the progeny male and female flies, male flies were used for the transcriptome analysis with and without a *M*. *luteus* infection. The expression of *CR44404* was tested in both female and male flies and found to be equivalent, after which male flies were used in all of the subsequent experiments.

For the lifespan experiment, *lincRNA-IBIN* overexpressing flies (*lincRNA-IBIN*^*1*^ and *lincRNA-IBIN*^*7*^ lines) were crossed with *C564*> driver flies at +25°C. After one day, the eggs were transferred to develop at +29°C for a maximum *lincRNA-IBIN* overexpression for the entire lifespan of the flies. Twice a week, the number of the flies was recorded and the flies transferred to fresh food.

### Culturing bacteria for infections

*Micrococcus luteus* (*M*. *luteus*) was cultured on Luria-Bertani (LB) agar plates under Streptomycin selection (final concentration 100 μg/ml) and left to grow at 29°C for 2–3 days. *Enterobacter cloacae* (*E*. *cloacae*) was cultured on LB agar plates under Nalidixic acid selection (final concentration 15 μg/ml) and the plates were incubated overnight at 37°C. The concentrated bacterial culture used for pricking the flies was prepared by collecting the colonies from the plate into 100μl of 50% glycerol in phosphate buffered saline (PBS; 137 mmol/l NaCl, 2.7 mmol/l KCl, 10 mmol/l Na_2_HPO_4_, 1.8 mmol/l KH_2_PO_4_).

*Enterococcus faecalis* (*E*. *faecalis*) was cultured in Brain-Heart-Infusion (BHI) medium and incubated at 37°C with shaking (225 rpm) overnight. The absorbance of the *E*. *faecalis* bacterial culture grown overnight was measured with a spectrophotometer at 600nm after which it was diluted 1:25 in 5 ml of BHI medium and left to grow for 2–3 hours at 37°C with shaking (225 rpm) until the absorbance at 600nm was 0.75. Then 2 ml of the bacterial culture was centrifuged at 700 x g for 5 min and the supernatant was discarded. The pellet was resuspended into 100 μl of 50% glycerol in PBS and the bacterial concentrate was used for pricking the flies.

### Fly infections

For bacterial infections, 0–2 day old male flies were collected and placed at +29°C for 48h, after which a septic injury to the flies was caused by pricking them in the thorax with a thin sharp tungsten wire dipped into a concentrated bacterial culture. For the activation of the Toll pathway, flies were infected with *M*. *luteus* (Gram-positive bacteria) and incubated for 24h at 25°C. For measuring AMP expression levels, infected flies and non-infected controls were incubated at 25°C for the duration of the infection, harvested, and their RNAs were extracted as described below. For survival experiments, *M*. *luteus* infected flies (24h, 25°C) were subsequently infected with *E*. *faecalis* and incubated at RT. The survival of the flies was monitored for 48h, as described earlier [39]. To activate the Imd pathway, the flies were infected with the Gram-negative bacterium *E*. *cloacae*, and the flies were incubated at 25°C for the duration of the infection.

### Infecting *Drosophila* larvae with *Leptopilina boulardi* wasps

2^nd^ instar larvae were infected with strain G486 of *L*. *boulardi* parasitoid wasps by placing 20 female wasps in vials with larvae. After two hours at room temperature, the wasps were removed and the larvae were transferred back to +29°C. 48 hours later the larvae were dissected to collect the hemocytes, plasma and fat bodies. The infection status of the larvae was checked by visually confirming the presence of *L*. *boulardi* eggs or larvae.

### Transcriptome analysis from total RNA (RNA sequencing)

For the first transcriptome analysis (Fig 1A and 1B), total RNAs from uninfected or *M*. *luteus* -infected (24h *p*.*i*.) *w*, *osa*^*IR*^ male flies were extracted with the TRI reagent. For the second transcriptome analysis (Fig 4), total RNAs were extracted from uninfected male flies with *lincRNA-IBIN* overexpression (*C564>lincRNA-IBIN*^*7*^), uninfected controls (*w*^*1118*^,*lincRNA-IBIN*^*7*^), *M*. *luteus* -infected *lincRNA-IBIN* OE and control flies (24 h *p*.*i*.) and *E*. *cloacae* -infected *lincRNA-IBIN* OE and control flies (6 h *p*.*i*.). All the sample groups were crossed at the same time and kept in the same conditions until collection. The resulting RNA samples were DNase treated with the RapidOut DNA removal kit (Thermo Scientific). The quality of the total RNA samples was ensured with the Advanced Analytical Fragment Analyzer and found to be good. Total RNA samples were pure, intact and all samples were of similar quality.

The preparation of the RNA libraries and Illumina HiSeq 2500 sequencing were carried out in the Finnish Microarray and Sequencing Centre (Turku, Finland). The RNA libraries were prepared according to the Illumina TruSeq Stranded mRNA Sample Preparation Guide (part # 15031047): Firstly, the poly-A containing RNA molecules were purified using a poly-T oligo attached to magnetic beads. Following purification, the RNA was fragmented into small pieces using divalent cations under an elevated temperature. The cleaved RNA fragments were copied into first strand cDNA using reverse transcriptase and random primers. Strand specificity was achieved by replacing dTTP with dUTP in the Second Strand, followed by second strand cDNA synthesis using DNA Polymerase I and RNase H. The incorporation of dUTP in second strand synthesis quenches the second strand during amplification, because the polymerase used in the assay is not incorporated past this nucleotide. The addition of Actinomycin D to First Stand Synthesis Act D mix (FSA) prevents spurious DNA-dependent synthesis, while allowing RNA-dependent synthesis, improving strand specificity. These cDNA fragments then have the addition of a single 'A' base and subsequent ligation of the adapter: the Unique Illumina TruSeq indexing adapter was ligated to each sample during the adapter ligation step for later pooling of several samples in one flow cell lane. The products were then purified and enriched with PCR to create the final cDNA library. Typically, the RNAseq library fragments are in the range of 200–700 bp and the average size of the fragments is 250–350 bp. The samples were normalized, pooled for the automated cluster preparation and sequenced with an Illumina HiSeq 2500 instrument using TruSeq v3 sequencing chemistry. Paired-end sequencing with a 1 x 50 bp read length was used, followed by a 6 bp index run. The technical quality of the HiSeq 2500 run was good and the cluster amount was as expected.

In both transcriptome analyses, the reads obtained were aligned against the *Drosophila melanogaster* reference genome (BDGP6 assembly, downloaded from the Illumina iGenomes website and originally derived from Ensembl). The reads were associated with known genes based on RefSeq annotations derived from UCSC database and the number of reads associated with each gene was counted using the featureCount method. The counts were normalized using the TMM normalisation method of the edgeR R/Bioconductor package. The number of reads is represented as RPKM values (Reads Per Kilobase of exon per Million reads mapped). RPKM = total gene reads / [mapped reads (millions) x total length of gene exons (kb)]. Genes with expression values (read number) of less than 0.125 across the treatments were considered to be expressed at low levels and excluded from the analysis.

### Tissue preparation for RNA extraction

For extracting RNA from whole flies or larvae, 3 x 5 individuals per phenotype were collected and snap-frozen on dry ice or in liquid nitrogen. For RNA extraction from the fat body, fat bodies from 3^rd^ instar larvae were dissected with forceps under a stereomicroscope and washed by dipping them three times into a 20 μl drop of 1 x PBS. In total, three biological replicates were prepared and pools of whole fat bodies from ten larvae per each biological replicate were used. Samples were snap-frozen in liquid nitrogen and stored at -80°C until RNA extraction. For RNA extraction from hemocytes and plasma, 55–60 larvae per replicate were washed, placed in a drop of 1 x PBS on a multiwell glass slide and dissected with forceps to release hemolymph. To separate hemocytes and plasma from hemolymph, suspensions were centrifuged at 2500 x g for 10 min, after which the plasma was carefully pipetted into a clean tube. Hemocytes and plasma samples were snap-frozen in liquid nitrogen and stored at -80°C until RNA extraction. For RNA extraction from adult guts, flies were dipped in 70% ethanol and dissected on a glass slide in 15 μl of 1 x PBS. The midgut region of the gut was separated and washed in a second drop of 1 x PBS. Guts from 10 flies per sample were pooled and centrifuged at 2000 x g for 2 min, after which PBS was removed and guts snap-frozen in liquid nitrogen and stored at -80°C until extraction.

### RNA extraction

To start the RNA extraction, a sufficient amount of the TRI reagent (MRC, Fisher Scientific) was added to the frozen whole flies, larvae or tissues. Whole flies, larvae, fat body and gut tissues were quickly thawed and homogenized in the TRI reagent using a micropestle (Fisher Scientific). Hemocytes were homogenized in the TRI reagent by pipetting up and down for a minimum of ten times. Plasma samples were quickly thawed and suspended in the TRIzol LS reagent (Thermo Fisher Scientific) by pipetting up and down ten times. Thereafter, total RNAs were extracted according to the manufacturer’s (TRI reagent or TRIzol LS) instructions. RNA pellets were dissolved in nuclease-free water, and the RNA concentrations and the purity were determined by a Nano-Drop 2000 (Thermo Scientific) measurement.

### Quantitative real-time PCR

Quantitative real-time PCR (qRT-PCR) was carried out with the iTaq Universal SYBR Green One-step kit (Bio-Rad, Hercules, CA, USA) using total RNAs (approximately 40 ng/sample) as templates. *RpL32* or *ND-39* was used as a housekeeping gene to normalize differences in RNA amounts between samples. In the experiments presented in Fig 3C, the amounts were standardized to 40 ng of total RNA/sample. This is because no products/mRNA from genes that are considered to have a housekeeping role are normally found in the plasma. Expression levels of genes in the test groups and controls were measured within the same qRT-PCR experiment. If the samples within an experiment did not fit in one 96-well plate, a reference sample was measured in all plates to make internal normalization between plates possible. In the qRT-PCR experimental figures, one uninfected control sample (indicated in the figure legend) was set to 1, to calculate fold-induction values. The primers used are listed in Table 1.

**Table 1:** qPCR primers.

Primer/ Gene	Forward, 5’→3’	Reverse, 5’→3’	product (bp)	Target
qRT-PCR primers				
*lincRNA-IBIN*	CAACTGCTGCCAATCCTCG	GCCTGGGATCGTAGTCACTT	103	qRT-PCR
*Drs*	ATGATGCAGATCAAGTACTTG	GCATCCTTCGCACCAGC	210	qRT-PCR
*IM1*	CTCGGTCTGCTGGCTGTGGC	CCGTGGACATTGCACACCC	95	qRT-PCR
*ND-39*	ACCGACAAGGTTCTGACTGG	CTCCGCTTAGGCAAACAGAC	201	qRT-PCR, control gene
*RpL32*	GGTTACGGATCGAACAAGCG	TTCTGCATGAGCAGGACCTC	101	qRT-PCR, control gene
*Lsp1alpha*	GCACTACACGCACTTCGATC	CCCAGTCCTTGGCGTAGTAG	158	qRT-PCR
*Hml*	TGCACCTGTAAGAACGGTCA	GATAATGCGGATCTCCAACG	82	qRT-PCR
*Mal-A1*	GACCGACGTCTGGATCAG	GTGAAGCCTGCTTTGGAG	138	qRT-PCR
*Mal-A8*	CACTGCCTCCGCTTTTTGAG	CGTGGTGGTCAGATAGTCGC	110	qRT-PCR
*epsilonTry*	AGTCGATTGAGGCCAAGGAC	CCATGGTGCGGGAGTTGTAG	120	qRT-PCR

### Quantification of larval hemocytes with flow cytometry

Individual 3^rd^ instar wasp-infected and uninfected *msnCherry*,*eaterGFP;C564>lincRNA-IBIN* (*MeC564>lincRNA-IBIN*), *msnCherry*, *eaterGFP; Hml*^*Δ*^*>; He > lincRNA-IBIN* (*MeHH>lincRNA-IBIN*) and control larvae were placed in a 20 μl drop of cold 8% BSA in 1 x PBS and dissected carefully with forceps. Carcasses were removed and the bled hemolymph was pipetted into a vial with 80 μl of 8% BSA in 1 x PBS. Ten larvae were dissected per cross and each cross was replicated three times. The samples were run with a BD Accuri C6 flow cytometer (BD, Franklin Lakes, NJ, USA), using a gating strategy established in [80]. In short, GFP-positive cells were detected in the FL1 (510/15 BP filter) and mCherry-positive cells in the FL3 (610/20 BP filter). GFP-only, mCherry-only and non-labelled hemocytes were used to establish the gates. Some of the GFP fluorescent signal was detected in the non-primary FL3 detector, and this was corrected for by subtracting 9% from the signal.

To check how well centrifuging separated the hemocyte and plasma fractions, five late 3^rd^ instar *HH>GFP* larvae were bled in 100 μl of 1 x PBS. The vials were centrifuged for 10 minutes at 2500 g at +4°C. The supernatant containing the plasma was pipetted into another vial (~90 μl) and the hemocyte pellet was re-suspended in 90 μl of 1 x PBS. Plasma and hemocyte samples were run with a flow cytometer and the numbers of GFP-positive hemocytes in both fractions were determined.

### Imaging of *Drosophila* larvae

Late 3^rd^ instar larvae were gently washed in a drop of water with a brush, dried on a piece of tissue paper and placed on a glass slide dorsal side facing up in a drop of 70% ice-cold glycerol. A coverslip was placed on the larvae and they were stored at +4°C overnight. The next day, the immobilized larvae were imaged with a Zeiss AxioImager M2 with Apotome 2, with an EC Plan Neofluar 5x/0.16 objective. A Colibri LED light source was used to excite GFP (LED 470 nm) and mCherry (LED 555 nm) and images were captured with an AxioCam HRm CCD camera. Images were processed with ImageJ (Version: 2.0.0-rc-59/1.51j) and Adobe Photoshop CS4. Ten larvae per cross were imaged.

### Cellular localization of *lincRNA-IBIN* with RNA FISH in the larval hemocytes

For detecting the cellular localization of *lincRNA-IBIN*, the RNA fluorescence in situ hybridization (RNA FISH) method with Cy3-tagged probes labeling the *lincRNA-IBIN* molecules was used. Late 3^rd^ instar male larvae were washed in a drop of water with a brush and the hemolymph of two larvae per sample type (four biological replicates) was carefully bled out from the larvae in 20 μl of ice-cold 1 x PBS on a multiwell glass slide well, avoiding contamination from other tissues. Hemocytes were left to adhere for one hour in a humidified chamber at RT. Samples were fixed with cold 3.7% paraformaldehyde in 1 x PBS for 5–10 min and washed with 1 x PBS for 3 x 5 min. Samples were permeabilized with 1 x PBS + 0.1% Triton X-100 for 5 minutes and washed with 1 x PBS until there was no foam, and the mask around the wells was dried carefully with a tissue paper. The samples were blocked with 3% BSA in 1 x PBS at +4°C o/n. The RNA FISH protocol was performed by using the QuantiGene ViewRNA Assay (Affymetrix) and the probes for *lincRNA-IBIN* and *RpL32* for *Drosophila* are now available in their catalog. For the hybridization of *lincRNA-IBIN* and *RpL32* probes (control) and a negative “no probe” control, pre-warmed diluents and humidified chambers were used, and the incubator temperature (+40°C) was monitored. The Working Probe Set Solution was prepared by diluting each probe set 1:100 in Probe Set Diluent QF: 20 μl drops were prepared for each sample by combining 0.2 μl of Probe Set and 19.8 μl of Probe Set diluent QF. The previous solution was aspirated from the wells and replaced with 20 μl of the Working Probe Set Solution and the samples were incubated in humidified chambers for three hours at +40°C. Working Probe Set Solution was aspirated and the wells were washed three times with Wash Buffer (this was used in all the washes). 20 μl of PreAmplifier Mix solution per sample was prepared by diluting PreAmplifier Mix 1:25 in Amplifier Diluent QF and added to samples and incubated at +40°C for 30 min. After washing three times, Amplifier Mix solution was prepared by diluting Amplifier mix 1:25 in pre-warmed Amplifier Diluent QF, added to the samples and incubated at +40°C for 30 min. After three washes, the Label Probe Mix Solution was prepared by diluting Label Probe Mix 1:25 in Label Probe diluent QF, added to the samples and incubated at +40°C for 30 min. Samples were washed three times and were left for 10 min in the wash buffer for the final wash. The samples were mounted with 20 μl of ProLong Gold Antifade Mountant with DAPI (Thermo Fisher Scientific). Cover glasses were pressed on and the slides were left to harden overnight in the dark, transferred to +4°C for a day and imaged.

The samples were imaged with a Zeiss LSM 780 confocal microscope with a Plan Apochromat 63 x/1.4 oil immersion objective. A pulsed diode laser was used to excite DAPI (405 nm) and a diode laser (561 nm) was used to excite Cy3 for imaging *lincRNA-IBIN* and *RpL32*. Images were captured using a Quasar spectral GaAsP PMT array detector and camera allowing fast spectral imaging. Images were processed with ImageJ (Version: 2.0.0-rc-59/1.51j) and Adobe Photoshop CS4.

### Measuring glucose, trehalose and glycogen from adult hemolymph

*lincRNA-IBIN* overexpressing (*C564>lincRNA-IBIN*^*7*^) and control flies (*w*^*1118*^,*lincRNA-IBIN*^*7*^) were allowed to eclose for 2 days, collected in fresh vials and kept at 29°C for two days prior to collecting the hemolymph. For experiments with infected and uninfected flies, *w*^*1118*^ flies were collected as above. *w*^*1118*^ flies were kept in fresh vials at 25°C for one day, after which half of them were infected by septic injury with a *E*. *cloacae* -contaminated needle. Flies were kept at 25°C for another 24 h prior to collecting the hemolymph. The hemolymph was collected by pricking the flies in the thorax with a thin sharp tungsten wire sterilized in 70% ethanol. Pools of 50 pricked flies were collected on ice in 0.5 ul microtubes with small holes punctured in them and placed in 1.5 μl microtubes. The flies were centrifuged at 5000 x g for 5 min, after which 0.8 μl of hemolymph was collected from the bottom of the 1.5 ml tube and diluted 1:100 in Trehalase Buffer (TB; 5 mM Tris pH 5.5, 137 mM NaCl, 2.7 mM KCl). The samples were snap-frozen in liquid nitrogen and stored at -80°C. Glucose and trehalose were analyzed using a colorimetric assay (Sigma Glucose (GO) assay kit, GACO20) based on the glucose oxidase (GO) enzyme following the protocol described in [81]. First, a trehalase stock was prepared by diluting 3 μl of porcine trehalase (Sigma-Aldrich; T8778-1UN) with 1 ml of TB. Samples were heat-inactivated for 5 min at 70°C, and divided into two 40 μl aliquots; one treated with an equal amount of trehalase stock to break down trehalose into free glucose, and the other left untreated by adding an equal amount of TB only. The samples were then incubated at 37°C overnight. Glucose standards were prepared by diluting 16 μl of a 1 mg/ml glucose stock solution with 84 μl of TB. 2-fold standard dilution curves were generated. Next morning, a 30 μl aliquot of each sample, the dilution series and a blank were loaded onto a 96-well plate and 100 μl of the GO reagent (GAGO20 Glucose assay kit, Sigma-Aldrich) was added. The plate was sealed with parafilm and incubated at 37°C for one hour. To stop the reaction, 100 μl of 12 N sulfuric acid (H_2_SO_4_) was added on the samples, after which the absorbance at 540 nm was measured using the Wallac Envision 2104 Multilabel Reader (PerkinElmer). The amount of glucose and trehalose (glucose + trehalose—glucose) in the samples were determined according to the glucose standard curve.

### C564-GAL4 expression in the gut

To verify that the *C564-GAL4* driver is expressed in the guts of adult flies, *C564>GFP* males and females were dissected in a drop of 1 x PBS and their guts were removed. The guts were checked for GFP expression using a stereomicroscope fluorescence adapter (NIGHTSEA, MA, USA) with a Royal blue LED (440–460 nm) for excitation and a 500 nm long-pass filter. Images were captured with Nikon DS-Fi2 camera.

### Statistical analyses

The first transcriptome analysis (Fig 1A and 1B) data was analyzed using the R package Limma. The package uses a modified t-test to generate an FDR (false discovery rate) corrected p-value (adjusted p-value) for each comparison. In the second transcriptome data analysis, the comparison between *lincRNA-IBIN* overexpression and controls (Fig 4A, S4 Table and S5 Table) was done using a two-tailed t-test (unequal variances assumed) with a 5% false discovery rate (FDR) correction using the Benjamini-Hochberg method [82]. In Fig 4B, genes that had a normalized read number >10 in the treatment of interest and an expression fold change >2 were included in the cluster analysis performed with the DAVID Bioinformatics resources 6.8 (https://david.ncifcrf.gov) [83,84] online tool. For Fig 4C and 4D, pairwise comparisons between uninfected control sample and different treatments were carried out using a two-tailed t-test assuming unequal variances.

Statistical analyses of gene expression by qRT-PCR results were carried out using a two-tailed t-test for two samples assuming equal variances. Statistical analyses of fly survival experiments were carried out using the log-rank (Mantel-Cox) test with Prism 6 (GraphPad). Data on hemocyte quantifications and types were plotted and analyzed with R version 3.3.2 (2016-10-31), Copyright 2015 The R Foundation for Statistical Computing. Data were analyzed using analysis of variance (ANOVA) followed by Tukey’s HSD post hoc test when requirements for normality and homoscedasticity were met, and in other cases a non-parametric Kruskal-Wallis rank sum test followed by Dunn’s post hoc test were applied. The level of statistical significance was established as p < 0.05.

## Supporting information

S1 Table Upregulated genes in *M*. *luteus* infected flies. Upregulated genes in response to a *Micrococcus luteus* infection in adult *D*. *melanogaster*. Genes were ranked based on > 18 fold change difference between uninfected controls and *M*. *luteus* infected flies (24h *p*.*i*.). The averages and standard deviations (SD) for the gene expression values are listed based on the number of reads obtained from the normalized RNA sequencing data. (S1 Table is related to Fig 1A).
(DOCX)

S2 Table Immune responsive lncRNA genes. Upregulated lncRNA-genes in response to a *Micrococcus luteus* infection in adult *D*. *melanogaster*. Genes were ranked based on > 3 fold change difference between *M*. *luteus* infected flies (24h *p*.*i*.) and age matched uninfected controls. Most of these lncRNA genes are less than 1 kb long and positioned in chromosomes two and three. The type of the lncRNA is categorized based on its genomic location to either intergenic (between genes) or overlapping (other gene/genes at the same locus). The averages and standard deviations (SD) for the lncRNA gene expression values are listed based on the number of reads obtained from the normalized RNA sequencing data. (S2 Table is related to Fig 1B).
(DOCX)

S3 Table Transcriptome profiling of the effects of IBIN overexpression (OE) on the known Toll pathway target genes in uninfected and *M*. *luteus* -infected flies. Fold changes (FC) were calculated by comparing the expression values of each of the treatments to uninfected controls. Overexpressing *lincRNA-IBIN* slightly increases the expression levels of *Drosomycin* (*Drs*) and *Immune induced molecules* (*IM*). Normalized expression values of the number of reads obtained from transcriptome sequencing are shown as the averages and standard deviations (SD). S3 Table is related to Fig 3C and 3D).
(DOCX)

S4 Table Genes upregulated in *lincRNA-IBIN* overexpressing flies. List of fold changes of genes that are significantly upregulated in *C564*>*lincRNA-IBIN* flies compared to control flies. Stars denote p-values from a two-tailed t-test that were significant after adjusting for a false discovery rate of 5%. *E*. *cloacae* and *M*. *luteus* columns show fold changes for *lincRNA-IBIN* -regulated genes in infected flies compared to uninfected control flies. Annotations are according to Flybase version Fb_2018_05. (S4 Table is related to Fig 4). p-values: *** < 0.001, ** <0.01, *< 0.05.
(DOCX)

S5 Table Genes downregulated in *lincRNA-IBIN* overexpressing flies. List of fold changes (FC) of genes that are significantly downregulated in *C564*>*lincRNA-IBIN* flies compared to control flies. Stars denote p-values that were significant after adjusting for a false discovery rate of 5%. *E*. *cloacae* and *M*. *luteus* columns show fold changes for *lincRNA-IBIN* -regulated genes in infected flies compared to uninfected control flies. Annotations are according to Flybase version Fb_2018_05. (S5 Table is related to Fig 4). p-values: *** < 0.001, ** <0.01, *< 0.05
(DOCX)

S1 Fig **Expression of A) *IM1* and B) *lincRNA-IBIN* upon a *M*. *luteus* infection (24) in male and female *Drosophila*.** *w*^*1118*^; *C564*> male and female flies were infected with *M*. *luteus* and collected 24h later with uninfected control flies. Gene expression levels were measured from total RNAs extracted from 3 biological replicates containing 5 flies each. (S1 Fig is related to Fig 1).
(TIF)

S2 Fig Predicted secondary structures of *lincRNA-IBIN*. **A)** The secondary structure for *lincRNA-IBIN* was predicted according to the lowest free energy structure for the sequence and **B)** composed based on the most probable base pairing, which is an alternative method that may have a higher fidelity in a structure prediction. Structure predictions were carried out with the RNAstructure -program (Web servers for RNA Secondary Structure Prediction) https://rna.urmc.rochester.edu/RNAstructureWeb/index.html.
(DOCX)

S3 Fig Detection of hemocytes in the plasma and hemocyte fractions. Hemolymph samples were centrifuged for 10 minutes at 2500g and the supernatant was pipetted into a separate vial. The plasma and hemocyte fractions were analysed with a BD Accuri C6 flow cytometer for the presence of hemocytes. **A)** A majority of GFP-positive hemocytes was detected in the hemocyte pellet fraction. **B)** Few hemocytes were seen in the plasma fraction. **C)** Numbers of hemocytes in the two fractions per pools of five *HH-GAL4 > GFP* larvae. (S3 Fig is related to Fig 2).
(PDF)

S4 Fig *lincRNA-IBIN* overexpression has no effect on the lifespan of flies. *lincRNA-IBIN* overexpressing flies (*lincRNA-IBIN*^*1*^ and *lincRNA-IBIN*^*7*^ lines) were crossed with *C564*> driver flies at +25°C and the eggs were transferred to +29°C for the entire lifespan of the flies. Twice a week, the number of flies was recorded and the flies transferred to fresh food. A) Lifespan of *C564*>*lincRNA-IBIN*^*7*^ flies and controls, B) lifespan of *C564*>*lincRNA-IBIN*^*1*^ flies and controls. (S4 Fig is related to Figs 3 and 4).
(TIF)

S5 Fig *lincRNA-IBIN* expression in the hemocytes causes an increase in hemocyte numbers in uninfected larvae, but does not affect hemocyte differentiation. Larvae were dissected with forceps in a drop of 8% BSA in 1 x PBS to release the hemolymph. **A)** Quantification of total hemocyte (eaterGFP and msnCherry positive hemocytes) and **A’)** lamellocyte counts (msnCherry-positive only) in larvae with *lincRNA-IBIN* expression in the hemocytes. **B)** Quantification of total hemocyte and **B’)** lamellocyte counts in larvae with *lincRNA-IBIN* expression in the fat body. *MeHH>* stands for *m**snCherry*, *e**aterGFP;* *H**ml*^*Δ*^*-GAL4;* *H**e-GAL4* and *MeC564> for* *msn**Cherry*, *e**aterGFP**; C564**-GAL4*. Dots represent individual larvae (10 larvae/replicate) and replicate crosses (three replicate crosses per genotype) are marked with different colors. Black bars represent the means. **C)** Representative images of whole larvae showing intact sessile bands. Scale bars 500 μm. Data were analyzed using ANOVA followed by Tukey’s HSD post hoc test or a non-parametric Kruskal-Wallis rank sum test followed by Dunn’s post hoc test. p-values smaller than 0.05 were considered significant.
(PDF)

S6 Fig Quantitative RT-PCR for selected genes was carried out with flies overexpressing *lincRNA-IBIN*^*7*^ or *lincRNA-IBIN*^*1*^ (*C564>*) and controls (*w*). **A)** Expression levels of *lincRNA-IBIN*; **B)** *Mal-A1* expression; **C)** *Mal-A8* expression; **D)** *epsilonTry* expression. Data were analyzed using a two-tailed t-test for two samples assuming equal variances. p-values smaller than 0.05 were considered significant. (S6 Fig is related to Fig 4).
(DOCX)

S7 Fig *lincRNA-IBIN* is expressed in the adult midgut upon a septic infection and modulates hemolymph glucose levels. **A)** the *C564-GAL4* driver is expressed in the *Drosophila* adult midgut, as demonstrated by *C564>GFP* expression. **B)** *lincRNA-IBIN* expression is strongly induced in the adult midgut of *C564>lincRNA-IBIN*^*7*^ flies. **C)** Hemolymph trehalose levels are not affected in *E*. *cloacae* -infected flies. (S7 Fig is related to Fig 4).
(DOCX)
