# Correction: Immune-inducible non-coding RNA molecule *lincRNA-IBIN* connects immunity and metabolism in *Drosophila melanogaster*

**DOI:** 10.1371/journal.ppat.1008088

**Published:** 2019-10-04

**Authors:** Susanna Valanne, Tiina S. Salminen, Mirva Järvelä-Stölting, Laura Vesala, Mika Rämet

The authors note that the annotation of two of the genes analyzed in their article has changed. These genes, that were previously annotated as non-coding genes, and analyzed accordingly, are now re-analyzed as coding genes. Therefore, the gene *lincRNA-IBIN* appears incorrectly throughout the article. The correct gene name is *IBIN*.

As a result, in the title of this article, the word “*lincRNA-IBIN*” should be “*IBIN*.” The correct title is: Immune-inducible *IBIN* connects immunity and metabolism in *Drosophila melanogaster*. The correct citation is: Valanne S, Salminen TS, Järvelä-Stölting M, Vesala L, Rämet M (2019) Immune-inducible *IBIN* connects immunity and metabolism in *Drosophila melanogaster*. PLoS Pathog 15(1): e1007504. https://doi.org/10.1371/journal.ppat.1007504

The authors have provided an additional explanation to provide context for these changes.

## Supporting information

S1 File IBIN comment 300819. (DOCX)
