# Supplementary material for: Immune-inducible non-coding RNA molecule lincRNA-IBIN connects immunity and metabolism in Drosophila melanogaster
Source: PLoS Pathog. 2019 Jan 11;15(1):e1007504. doi: 10.1371/journal.ppat.1007504 (PMC6345493; doi:10.1371/journal.ppat.1007504)
Supplement: S1 Table — Upregulated genes in response to a Micrococcus luteus infection in adult D. melanogaster. Genes were ranked based on > 18 fold change difference between uninfected controls and M. luteus infected flies (24h p.i.). The averages and standard deviations (SD) for the gene expression values are listed based on the number of reads obtained from the normalized RNA sequencing data. (S1 Table is related to Fig 1A). (DOCX) [file ppat.1007504.s001.docx]

**S1 Table**

| **Gene symbol** | **Gene name** | **Flybase ID** | **Fold change** | **Adj. p-value** | **Uninfected average ± SD** | **Infected**  **average ± SD** | **Annotation** |
| --- | --- | --- | --- | --- | --- | --- | --- |
| *CR44404* | *lincRNA-IBIN* | FBgn0265577 | 1288.9 | 5,6E-06 | 0.97 ± 0.26 | 1500 ± 398.9 | lncRNA gene |
| *SPH93* | *Serine protease homolog 93* | FBgn0032638 | 1085.1 | 2,0E-08 | 0.27 ± 0.09 | 317.4 ± 35.4 | Defense response to Gram-positive bacterium |
| *CG33462* | *CG33462* | FBgn0053462 | 546.5 | 3,1E-04 | 0 | 25.4 ± 8.3 | Serine-type endopeptidase activity |
| *Dro* | *Drosocin* | FBgn0010388 | 417.7 | 1,2E-09 | 4.5 ± 0.7 | 1924.4 ± 223.3 | Antibacterial humoral response |
| *Mtk* | *Metchnikowin* | FBgn0014865 | 330.8 | 1,8E-08 | 13.5 ± 2.8 | 4460.6 ± 1025.8 | Antibacterial humoral response, defense response to fungus |
| *CG4757* | *CG4757* | FBgn0027584 | 294.8 | 5,2E-14 | 8.5 ± 1.0 | 2486.8 ± 38.3 | Carboxylic ester hydrolase activity |
| *CG30091* | *CG30091* | FBgn0050091 | 274.1 | 5,4E-05 | 0.1 ± 0.05 | 19.8 ± 2.1 | Serine-type endopeptidase activity |
| *Drs* | *Drosomycin* | FBgn0010381 | 265.1 | 5,2E-14 | 45.1 ± 7.7 | 11733.1 ±548.8 | Defense response to Gram-negative bacterium, defense response to fungus |
| *IM23* | *Immune inducible molecule 23* | FBgn0034328 | 121.4 | 1,6E-11 | 20.1 ± 3.9 | 2373.8 ± 222.8 | Defense response to Gram-positive bacterium, Toll signaling pathway |
| *CG18563* | *CG18563* | FBgn0032639 | 117.4 | 1,0E-06 | 0.3 ± 0.4 | 26.3 ± 1.8 | Proteolysis (adjacent to the *SPH93* in the genome) |
| *CG11459* | *CG11459* | FBgn0037396 | 91.6 | 1,2E-08 | 1.3 ± 0.6 | 112.3 ± 8.6 | Aminopeptidase activity, immune response |
| *IM1* | *Immune induced molecule 1* | FBgn0034329 | 91.5 | 1,6E-11 | 77.9 ± 10.8 | 7059.6 ± 907.6 | Defense response to Gram-positive bacterium |
| *CG43202* | *CG43202* | FBgn0262838 | 81.2 | 2,0E-08 | 3.1 ± 0.5 | 264.8 ± 30.1 | na |
| *CR45045* | *CR45045* | FBgn0266405 | 64.6 | 6,3E-04 | 0.1 ± 0.2 | 17.3 ± 5.5 | lncRNA gene, na |
| *Sid* | *Stress induced DNase* | FBgn0039593 | 53.6 | 1,5E-08 | 1.1 ± 0.4 | 55.4 ± 2.0 | Induced by bacterial infection and oxidative stress, nucleic acid phosphodiester bond hydrolysis |
| *GNBP-like 3* | *GNBP-like 3* | FBgn0034511 | 43.6 | 3,6E-11 | 38.8 ± 7.8 | 1662.1 ± 190.5 | Defense response to other organisms, response to fungus |
| *IM3* | *Immune induced molecule 3* | FBgn0040736 | 43.1 | 2,7E-10 | 107.3 ± 24.5 | 4604.7 ± 173.6 | Defense response to Gram-positive bacterium, Toll signaling pathway |
| *CG43291* | *CG43291* | FBgn0262983 | 41.3 | 2,2E-04 | 0.01 ± 0.02 | 1,27 ± 0.25 | na |
| *DptB* | *Diptericin B* | FBgn0034407 | 38.1 | 6,4E-06 | 4.6 ± 1.0 | 178.4 ± 30.5 | Defense response to Gram-positive bacterium |
| *CG15065* | *CG15065* | FBgn0040734 | 34.2 | 2,6E-07 | 44.8 ± 7.0 | 1554.0 ± 472.5 | Defense response |
| *AttC* | *Attacin-C* | FBgn0041579 | 34.0 | 0,5E-03 | 6.6 ± 0.7 | 222.9 ± 17.9 | Antibacterial humoral response |
| *AttA* | *Attacin-A* | FBgn0012042 | 31.2 | 4,6E-04 | 1.9 ± 0.3 | 58.8 ± 7.4 | Antibacterial humoral response |
| *IM2* | *Immune induced molecule 2* | FBgn0025583 | 30.6 | 1,7E-11 | 267.2 ± 19.2 | 8171.4 ± 737.5 | Response to bacterium |
| *IM14* | *Immune induced molecule 14* | FBgn0067905 | 27.6 | 3,6E-11 | 253.7 ± 14.6 | 7112.9 ± 1249.1 | Response to bacterium |
| *CG18067* | *CG18067* | FBgn0034512 | 27.0 | 6,0E-14 | 337.8 ± 7.8 | 9126.1 ± 486.4 | na |
| *CG5791* | *CG5791* | FBgn0040582 | 24.4 | 2,8E-11 | 15.1 ± 1.2 | 370 ± 32.2 | na |
| *IM4* | *Immune induced molecule 4* | FBgn0040653 | 23.1 | 1,4E-10 | 496.8 ± 95.7 | 11376.6 ± 391.0 | Response to bacterium |
| *CecA2* | *Cecropin A2* | FBgn0000277 | 18.9 | 2,5E-05 | 2.2 ± 0.4 | 43.5 ± 6.7 | Antibacterial humoral response |
