# Supplementary material for: Immune-inducible non-coding RNA molecule lincRNA-IBIN connects immunity and metabolism in Drosophila melanogaster
Source: PLoS Pathog. 2019 Jan 11;15(1):e1007504. doi: 10.1371/journal.ppat.1007504 (PMC6345493; doi:10.1371/journal.ppat.1007504)
Supplement: S2 Table — Upregulated lncRNA-genes in response to a Micrococcus luteus infection in adult D. melanogaster. Genes were ranked based on > 3 fold change difference between M. luteus infected flies (24h p.i.) and age matched uninfected controls. Most of these lncRNA genes are less than 1 kb long and positioned in chromosomes two and three. The type of the lncRNA is categorized based on its genomic location to either intergenic (between genes) or overlapping (other gene/genes at the same locus). The averages and standard deviations (SD) for the lncRNA gene expression values are listed based on the number of reads obtained from the normalized RNA sequencing data. (S2 Table is related to Fig 1B). (DOCX) [file ppat.1007504.s002.docx]

**S2 Table**

| **lncRNA gene** | **Flybase ID** | **Fold change** | **Adj. p-value** | **Uninfected average ± SD** | **Infected**  **average ± SD** | **Length (nc)** | **Genomic location** | **Type** |
| --- | --- | --- | --- | --- | --- | --- | --- | --- |
| *CR44404* | FBgn0265577 | 1288.9 | 5,6E-06 | 0,97 ± 0,26 | 1500,0± 398,9 | 228 | 2R [+] | intergenic |
| *CR45045* | FBgn0266405 | 64.6 | 6,3E-04 | 0,12 ± 0,21 | 17,29 ± 5,45 | 249 | 3R[+] | overlapping |
| *CR45570* | FBgn0267130 | 9.0 | 6,5E-03 | 0,36 ± 0,23 | 3,62 ± 0,97 | 561 | 3R [+] | intergenic |
| *CR45585* | FBgn0267145 | 7.3 | 1,7E-02 | 0,49 ± 0,41 | 3,79 ± 1,19 | 5851 | 3R[+] | intergenic |
| *CR44779* | FBgn0266005 | 6.4 | 1,4E-08 | 1,60 ± 0,20 | 6,66 ± 0,59 | 41625 | X [+] | overlapping |
| *CR45555* | FBgn0267115 | 6.3 | 3,0E-02 | 0,08 ± 0,07 | 0,84 ± 0,27 | 698 | 3R [+] | intergenic |
| *CR30009* | FBgn0050009 | 5.4 | 2,3E-02 | 0,04 ± 0,04 | 0,26 ± 0,14 | 2513 | 2R[-] | intergenic |
| *CR44366* | FBgn0265497 | 4.9 | 3,4E-05 | 0,72 ± 0,28 | 3,56 ± 0,44 | 1591 | 2R [+] | intergenic |
| *CR45269* | FBgn0266807 | 4.3 | 1,3E-02 | 0,25 ± 0,07 | 1,45 ± 0,49 | 481 | 2R [-] | overlapping |
| *CR43992* | FBgn0264724 | 4.1 | 4,7E-02 | 0,07 ± 0,12 | 0,65 ± 0,11 | 1390 | 3L [+] | intergenic |
| *CR44964* | FBgn0266279 | 3.6 | 1,4E-03 | 0,26 ± 0,08 | 0,99 ± 0,27 | 1622 | X [-] | overlapping |
| *CR44320* | FBgn0265379 | 3.6 | 2,9E-02 | 0,31 ± 0,29 | 1,16 ± 0,11 | 35019 | 3R [+] | intergenic |
| *CR44932* | FBgn0266237 | 3.4 | 4,0E-02 | 0,07 ± 0,06 | 0,35 ± 0,04 | 951 | 3R [-] | intergenic |
| *CR44268* | FBgn0265254 | 3.4 | 6,7E-03 | 1,24 ± 0,39 | 4,83 ± 1,25 | 393 | 2L [+] | overlapping |
| *CR45022* | FBgn0266380 | 3.1 | 9,2E-03 | 0,86 ± 0,55 | 2,96 ± 0,31 | 318 | 2R [+] | overlapping |
| *CR45410* | FBgn0266959 | 3.0 | 2,3E-02 | 0,24 ± 0,13 | 0,78 ± 0,34 | 1182 | 3L [+] | intergenic |
