# Supplementary material for: Immune-inducible non-coding RNA molecule lincRNA-IBIN connects immunity and metabolism in Drosophila melanogaster
Source: PLoS Pathog. 2019 Jan 11;15(1):e1007504. doi: 10.1371/journal.ppat.1007504 (PMC6345493; doi:10.1371/journal.ppat.1007504)
Supplement: S3 Table — Fold changes (FC) were calculated by comparing the expression values of each of the treatments to uninfected controls. Overexpressing lincRNA-IBIN slightly increases the expression levels of Drosomycin (Drs) and Immune induced molecules (IM). Normalized expression values of the number of reads obtained from transcriptome sequencing are shown as the averages and standard deviations (SD). S3 Table is related to Fig 3C and 3D). (DOCX) [file ppat.1007504.s003.docx]

**S3 Table**

| **Uninfected control** | | ***IBIN* OE** | | ***M. luteus* infected** | | ***M. luteus* + *IBIN* OE** | |
| --- | --- | --- | --- | --- | --- | --- | --- |
| **Gene ID** | **Average ± SD** | **Average ± SD** | **FC** | **Average ± SD** | **FC** | **Average ± SD** | **FC** |
| *Drs* | 16,8 ± 6,7 | 19,7 ± 6,4 | **1,2** | 11621,2 ± 644,6 | **691,8** | 13019,3 ± 428,6 | **775,0** |
| *IM23* | 15,5 ± 3,9 | 25,7 ± 4,8 | **1,7** | 2346,1 ± 256,6 | **151,1** | 2933,6 ± 397,3 | **189,0** |
| *IM1* | 55,5 ± 6,1 | 84,0 ± 3,1 | **1,5** | 5507,6 ± 562,1 | **99,1** | 6656,5 ± 676,4 | **119,8** |
| *IM2* | 232,6± 10,6 | 483,1 ± 64,3 | **2,1** | 7938,5 ± 838,0 | **34,1** | 10400,0 ± 661,6 | **44,7** |
| *IM3* | 97,6 ± 11,7 | 137,0 ± 4,9 | **1,4** | 3083,0 ± 304,4 | **31,6** | 3330,4 ± 227,2 | **34,1** |
| *IM14* | 160,6± 17,4 | 241,0 ± 15,3 | **1,5** | 4262,8 ± 278,6 | **26,6** | 4647,9 ± 425,2 | **28,9** |
| *IM4* | 218,1± 35,6 | 326,9 ± 38,5 | **1,5** | 5747,2 ± 458,2 | **26,4** | 6390,1 ± 366,1 | **29,3** |
