# Supplementary material for: Immune-inducible non-coding RNA molecule lincRNA-IBIN connects immunity and metabolism in Drosophila melanogaster
Source: PLoS Pathog. 2019 Jan 11;15(1):e1007504. doi: 10.1371/journal.ppat.1007504 (PMC6345493; doi:10.1371/journal.ppat.1007504)
Supplement: S4 Table — List of fold changes of genes that are significantly upregulated in C564>lincRNA-IBIN flies compared to control flies. Stars denote p-values from a two-tailed t-test that were significant after adjusting for a false discovery rate of 5%. E. cloacae and M. luteus columns show fold changes for lincRNA-IBIN -regulated genes in infected flies compared to uninfected control flies. Annotations are according to Flybase version Fb_2018_05. (S4 Table is related to Fig 4). p-values: *** < 0.001, ** <0.01, *< 0.05. (DOCX) [file ppat.1007504.s004.docx]

| **Gene** | ***lincRNA-IBIN* OE** | ***E. cloacae*** | ***M. luteus*** | **Annotation** |
| --- | --- | --- | --- | --- |
| *CR44404* | *165.9*** | *710.9** | *202.4* | *Immune inducible long non-coding RNA gene* |
| *Hsp70Bb* | 32.9*** | 3.7 | 1.1 | Response to unfolded protein; heat shock protein binding |
| *Npc2e* | 29.6* | 22.7 | 11.6 | Lipid antigen-, lipopolysaccharide-, peptidoglycan binding |
| *Amyrel* | 6.8* | -1.9 | -1.4 | Amylase activity; carbohydrate metabolic process |
| *Lsp2* | 5.9* | -1.2 | -3.7 | Motor neuron axon guidance; synaptic target inhibition |
| *CG17751* | 5.1*** | -1.2 | -1.1 | Transmembrane transport |
| *CG34040* | 4.9* | 7.0 | 5.1 | Non-annotated protein coding gene |
| *Ugt86Dd* | 3.6** | 1.1 | 1.0 | Glucuronosyltransferase activity |
| *CG15263* | 3.6* | 2.0* | 2.8* | Non-annotated protein coding gene |
| *CG32284* | 3.4** | 12.4 | 1.7 | Chitin binding; chitin metabolic process |
| *CG1139* | 3.0** | 3.3** | 2.3 | Amino acid transmembrane transport |
| *w* | 2.9*** | -1.1 | 1.0 | Transmembrane transport activity; compound eye pigmentation |
| *CG6277* | 2.9* | 1.3 | 1.1 | Lipase activity; lipid catabolic process |
| *Try29F* | 2.8* | -1.3 | -1.4 | Serine-type endopeptidase activity; proteolysis |
| *CG4734* | 2.8** | 1.2 | 1.4 | Non-annotated protein coding gene |
| *CG12780* | 2.8*** | 1.7* | 2.0** | Carbohydrate binding; defense response to virus |
| *CG33511* | 2.6* | 1.5 | -1.6 | Non-annotated protein coding gene |
| *tobi* | 2.6** | 2.3** | 2.1* | Carbohydrate metabolic process; glycoside catabolic process |
| *CG15818* | 2.6*** | 4.0** | 3.6 | Carbohydrate binding |
| *CG15533* | 2.5** | 1.2 | -1.2 | Ceramide biosynthetic process; sphingomyelin catabolic process |
| *CG13324* | 2.5** | 7.4 | 6.0* | Non-annotated protein coding gene |
| *CR44107* | 2.4* | 1.0 | -1.3 | Antisense RNA |
| *Cyp309a1* | 2.3*** | 1.7 | 1.0 | Oxidation-reduction process; heme binding |
| *Cyp6g1* | 2.3*** | -1.7** | -1.2* | Oxidoreductase activity; heme binding; response to insecticide |
| *CG43235* | 2.3 | 1.4 | 2.2 | Metallocarboxypeptidase activity; proteolysis |
| *CG5791* | 2.3** | 3.9** | 35.8* | Non-annotated protein coding gene |
| *CG30054* | 2.2*** | 1.1 | 1.6 | G-protein coupled receptor binding |
| *CG31148* | 2.2* | 1.4 | 1.1 | Glucosylceramidase activity; hydrolase activity |
| *Cpr67Fb* | 2.2** | 2.0 | -1.2 | Structural constituent of cuticle; chitin-based cuticle development |
| *CG30016* | 2.2** | -1.7** | -1.2 | Hydroxyisourate hydrolase activity; purine nucleobase metabolic process |
| *CG1208* | 2.2*** | 2.2*** | 1.8*** | Glucose transmembrane transporter activity; hexose transmembrane transport |
| *CG3819* | 2.1** | 4.2* | 3.4 | Endonuclease activity; DNA catabolic process |
| *Cyp4p1* | 2.1*** | 1.7** | 1.2 | Oxidation-reduction process; heme binding |
| *CG15043* | 2.1* | 7.1* | 5.6*** | Non-annotated protein coding gene |
| *Mal-A8* | 2.1** | 1.7* | 1.6 | Carbohydrate metabolic process; maltose alpha-glucosidase activity |
| *CG17562* | 2.1* | 2.0** | -1.4** | Long-chain fatty-acyl-CoA metabolic process |
| *CG17322* | 2.1*** | -1.3* | -1.2 | Hexosyl group transferase activity; UDP-glucose metabolic process |
| *IM2* | 2.1* | 14.1** | 34.1* | Defense response to bacterium<http://flybase.org/reports/GO:0035336> |
| *Mal-A1* | 2.1*** | 1.7** | 1.4 | Carbohydrate metabolic process; maltose alpha-glucosidase activity |
| *Mal-A7* | 2.1** | 1.6 | 1.6 | Carbohydrate metabolic process; maltose alpha-glucosidase activity |
| *Mal-A2* | 2.1** | 1.6** | 1.3** | Carbohydrate metabolic process; maltose alpha-glucosidase activity |
| *Sodh-1* | 2.1** | -5.9*** | -1.6 | Oxidation-reduction process |
| *Mal-B1* | 2.0** | 1.0 | 1.1 | Carbohydrate metabolic process; maltose alpha-glucosidase activity |
| *CR43414* | 2.0** | 1.0 | 1.2 | spermatogenesis |
| *CG15067* | 2.0** | 3.6** | 13.8 | Immune-induced protein Dim domain |

**S4 Table**
