# Supplementary material for: Immune-inducible non-coding RNA molecule lincRNA-IBIN connects immunity and metabolism in Drosophila melanogaster
Source: PLoS Pathog. 2019 Jan 11;15(1):e1007504. doi: 10.1371/journal.ppat.1007504 (PMC6345493; doi:10.1371/journal.ppat.1007504)
Supplement: S5 Table — List of fold changes (FC) of genes that are significantly downregulated in C564>lincRNA-IBIN flies compared to control flies. Stars denote p-values that were significant after adjusting for a false discovery rate of 5%. E. cloacae and M. luteus columns show fold changes for lincRNA-IBIN -regulated genes in infected flies compared to uninfected control flies. Annotations are according to Flybase version Fb_2018_05. (S5 Table is related to Fig 4). p-values: *** < 0.001, ** <0.01, *< 0.05 (DOCX) [file ppat.1007504.s005.docx]

| **Gene** | ***lincRNA-IBIN* OE** | ***E. cloacae*** | ***M. luteus*** | **Annotations** |
| --- | --- | --- | --- | --- |
| *epsilonTry* | -5.3 | 1.4 | -1.1 | Serine-type endopeptidase activity; proteolysis |
| *betaTry* | -3.2** | 1.0 | 1.0 | Serine-type endopeptidase activity; proteolysis |
| *Sfp33A4* | -3.2*** | -1.4 | -1.8 | Multicellular organism reproduction |
| *CG44088* | -3.1 | 1.6 | 1.3 | Non-annotated protein coding gene |
| *LysP* | -2.5** | -1.5 | -1.3 | Defense response to gram-negative bacterium; lysozyme activity |
| *CG17192* | -2.5 | -1.6 | -1.6 | Lipid catabolic process; lipase activity |
| *Jon65Ai* | -2.5 | -2.4 | -4.7 | Serine-type endopeptidase activity; proteolysis |
| *CG12374* | -2.3 | -1.4 | -1.8 | Metallocarboxypeptidase activity; proteolysis |
| *CG11911* | -2.3 | -1.5 | -1.3 | Serine-type endopeptidase activity; proteolysis |
| *CR43358* | -2.3** | -1.2 | -2.6** | Non-annotated long non-coding RNA gene |
| *CR43304* | -2.2** | -1.3 | -1.2 | Non-annotated long non-coding RNA gene |
| *CR45237* | -2.2 | -1.8 | -1.6 | Non-annotated long non-coding RNA gene |
| *Spn28Da* | -2.2** | -1.3 | -1.8** | Serine-type endopeptidase inhibitor activity; negative reg. of proteolysis |
| *CG3106* | -2.1 | -6.5 | -1.2 | Transferase activity; transferring acyl groups other than amino-acyl groups |
| *CG34211* | -2.1* | -1.2 | 1.3 | Non-annotated protein coding gene |
| *Acp76A* | -2.1** | 1.0 | -1.5 | Serine-type endopeptidase inhibitor activity; negative reg. of proteolysis |
| *Acp36DE* | -2.1** | 1.1 | 1.7** | Multicellular organism reproduction |
| *Jon65Aii* | -2.1* | -5.7 | -3.4** | Serine-type endopeptidase activity; proteolysis |
| *Npc2d* | -2.0 | -3.5 | -2.0 | Sterol binding; sterol transport |
| *CG43441* | -2.0* | -1.4 | -1.2 | Non-annotated protein coding gene |
| *CG17097* | -2.0** | -1.1 | -1.7** | Lipid catabolic process; lipase activity |

**S5 table**
