# Supplementary figures and images for: Immune-inducible non-coding RNA molecule lincRNA-IBIN connects immunity and metabolism in Drosophila melanogaster
Source: PLoS Pathog. 2019 Jan 11;15(1):e1007504. doi: 10.1371/journal.ppat.1007504 (PMC6345493; doi:10.1371/journal.ppat.1007504)

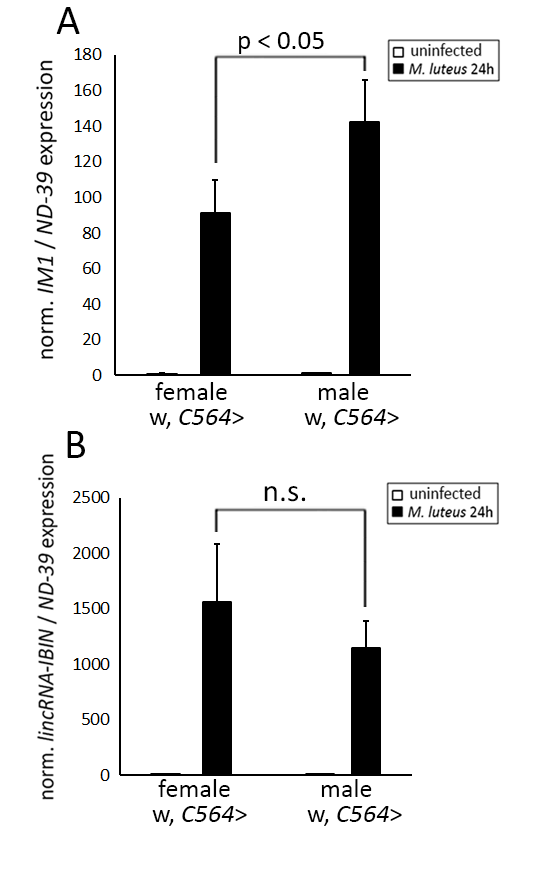

Supplement: S1 Fig — Expression of A) IM1 and B) lincRNA-IBIN upon a M. luteus infection (24) in male and female Drosophila. w1118; C564> male and female flies were infected with M. luteus and collected 24h later with uninfected control flies. Gene expression levels were measured from total RNAs extracted from 3 biological replicates containing 5 flies each. (S1 Fig is related to Fig 1). (TIF) [file ppat.1007504.s006.tif]

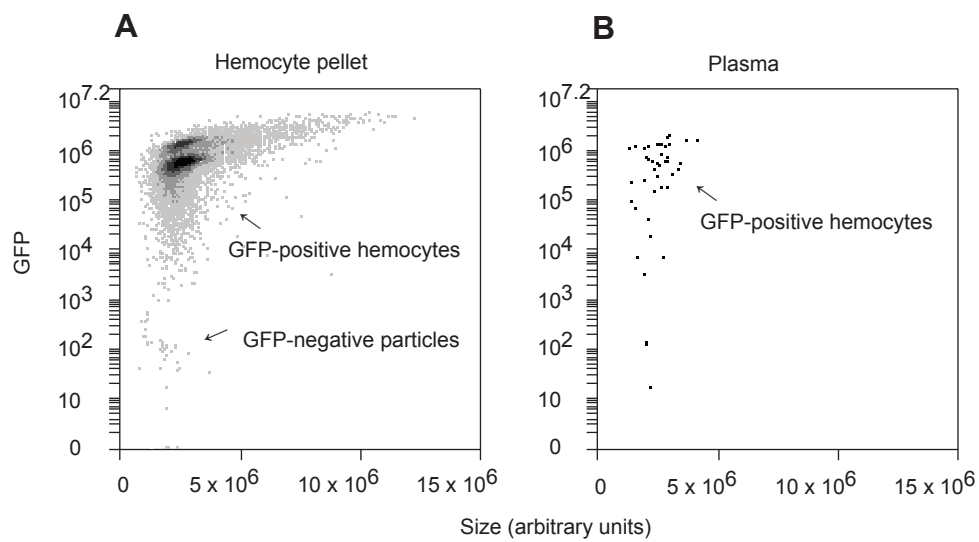

**C**

| Fraction  | Sample 1 | Sample 2 | Sample 3 |
|-----------|----------|----------|----------|
| Hemocytes | 34074    | 44010    | 63729    |
| Plasma    | 279      | 246      | 108      |

Supplement: S3 Fig — Hemolymph samples were centrifuged for 10 minutes at 2500g and the supernatant was pipetted into a separate vial. The plasma and hemocyte fractions were analysed with a BD Accuri C6 flow cytometer for the presence of hemocytes. A) A majority of GFP-positive hemocytes was detected in the hemocyte pellet fraction. B) Few hemocytes were seen in the plasma fraction. C) Numbers of hemocytes in the two fractions per pools of five HH-GAL4 > GFP larvae. (S3 Fig is related to Fig 2). (PDF) [file ppat.1007504.s008.pdf]

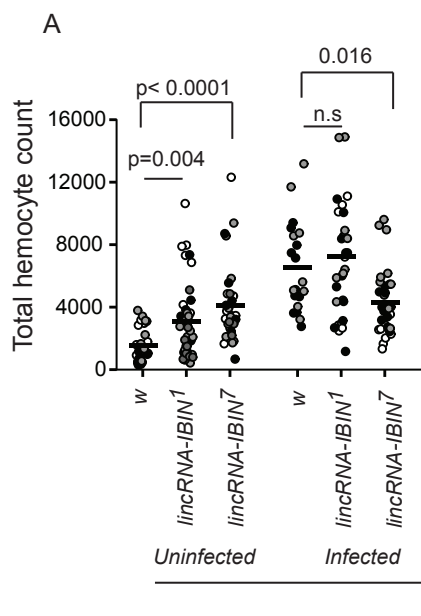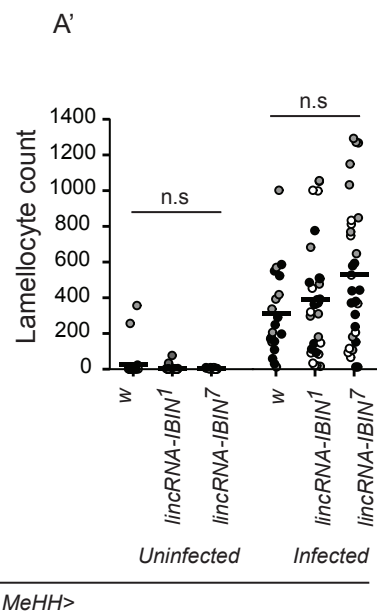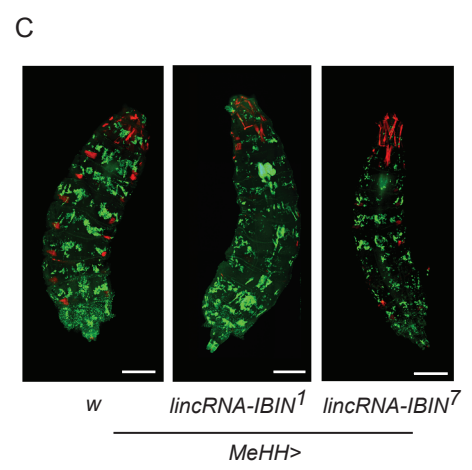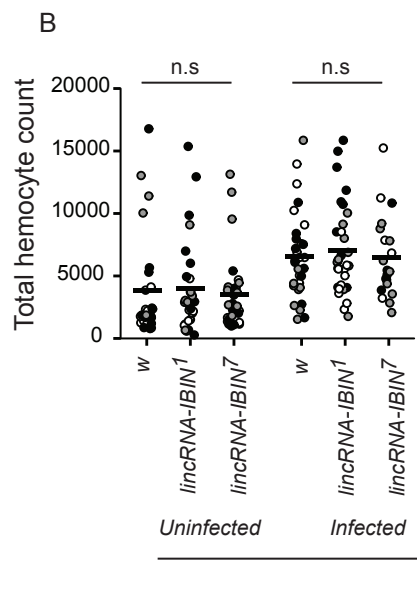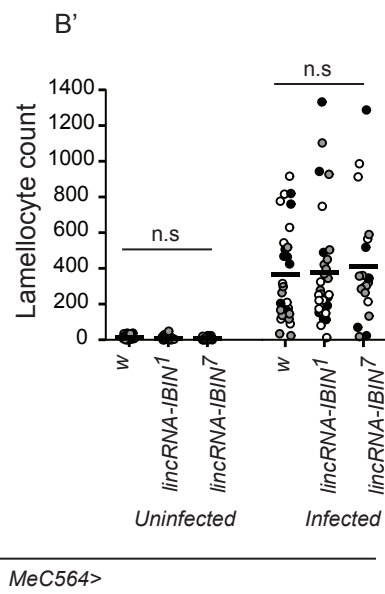

Supplement: S5 Fig — Larvae were dissected with forceps in a drop of 8% BSA in 1 x PBS to release the hemolymph. A) Quantification of total hemocyte (eaterGFP and msnCherry positive hemocytes) and A’) lamellocyte counts (msnCherry-positive only) in larvae with lincRNA-IBIN expression in the hemocytes. B) Quantification of total hemocyte and B’) lamellocyte counts in larvae with lincRNA-IBIN expression in the fat body. MeHH> stands for msnCherry, eaterGFP; HmlΔ-GAL4; He-GAL4 and MeC564> for msnCherry, eaterGFP; C564-GAL4. Dots represent individual larvae (10 larvae/replicate) and replicate crosses (three replicate crosses per genotype) are marked with different colors. Black bars represent the means. C) Representative images of whole larvae showing intact sessile bands. Scale bars 500 μm. Data were analyzed using ANOVA followed by Tukey’s HSD post hoc test or a non-parametric Kruskal-Wallis rank sum test followed by Dunn’s post hoc test. p-values smaller than 0.05 were considered significant. (PDF) [file ppat.1007504.s010.pdf]

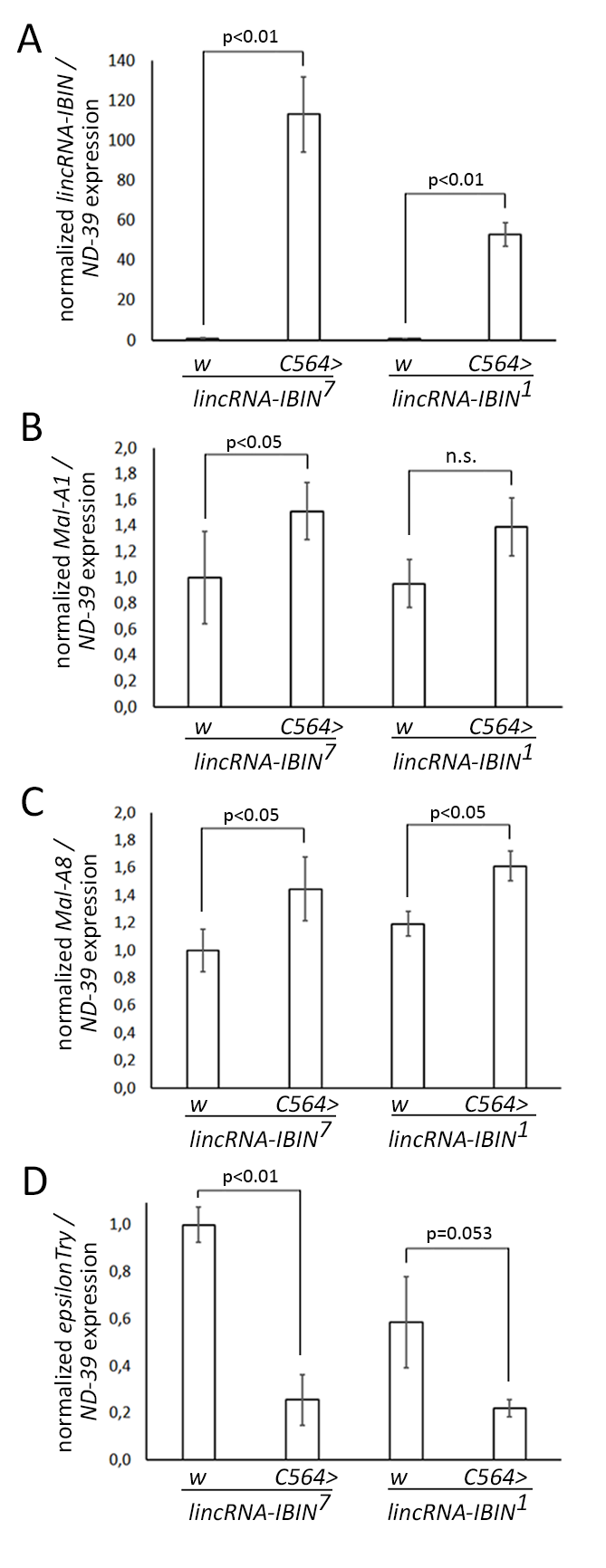


**S6 Figure**

Supplement: S6 Fig — A) Expression levels of lincRNA-IBIN; B) Mal-A1 expression; C) Mal-A8 expression; D) epsilonTry expression. Data were analyzed using a two-tailed t-test for two samples assuming equal variances. p-values smaller than 0.05 were considered significant. (S6 Fig is related to Fig 4). (DOCX) [file ppat.1007504.s011.docx]

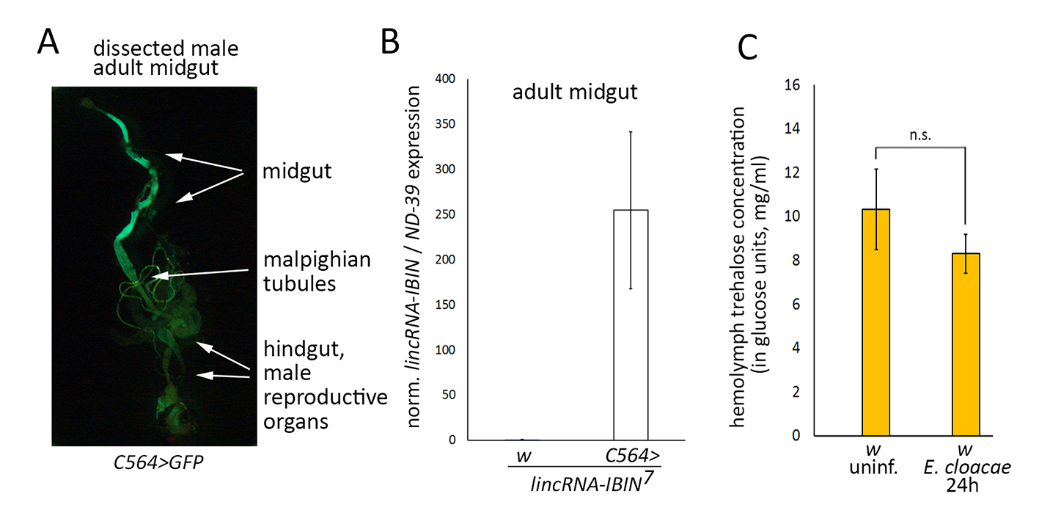


**S7 Figure**

Supplement: S7 Fig — A) the C564-GAL4 driver is expressed in the Drosophila adult midgut, as demonstrated by C564>GFP expression. B) lincRNA-IBIN expression is strongly induced in the adult midgut of C564>lincRNA-IBIN7 flies. C) Hemolymph trehalose levels are not affected in E. cloacae -infected flies. (S7 Fig is related to Fig 4). (DOCX) [file ppat.1007504.s012.docx]
