# Supplementary material for: Correction: Immune-inducible non-coding RNA molecule lincRNA-IBIN connects immunity and metabolism in Drosophila melanogaster
Source: PLoS Pathog. 2019 Oct 4;15(10):e1008088. doi: 10.1371/journal.ppat.1008088 (PMC6777747; doi:10.1371/journal.ppat.1008088)
Supplement: S1 File — (DOCX) [file ppat.1008088.s001.docx]

In this article (Valanne et al. 2019), we identified and characterized putative infection-inducible long non-coding RNA genes in *Drosophila*. However, on the contrary to earlier annotations at Flybase, based on recent reanalysis, we have come to the very likely conclusion, that two of the presumed long non-coding genes studied in this article, namely *CR44404* / *lincRNA:IBIN* and *CR45045*, are both protein-coding genes.

The gene names are changed accordingly: *lncRNA:IBIN* (*CR44404*) to *IBIN* (*CG44404*) and *lncRNA:CR45045* to *CG45045*. These two genes encode putative short polypeptides that are closely related to each other. Based on assessment of tBLASTn results using the FlyBase BLAST tool, at least one region encoding a similar polypeptide is found in most of the Sophophora species for which genomic sequence information is available.

Because of these changes, in our experiments in this article we have studied *IBIN* mRNA instead of a gene with a long non-coding RNA status. Nevertheless, results of the studies of *IBIN* expression and effects of *IBIN* overexpression remain valid. In the light of the changed nature of the gene products and the similarities between the polypeptides encoded by *CG44404* and *CG45045*, further research is required. We expect that both gene products are secreted, as both genes have a likely signal sequence at the 5’-end. The exact molecular function remains to be determined, as well as the definite proof of the existence of translated peptides.

IBIN peptide sequence: MQLLPILVLLALLICAACKNHEDWGGYRPSDYDPRPYFRQF
CG45045 peptide sequence: MRFLTVAVMLALVICAACKNHEEWKGQRPWDYDRRPPSNPYA

Respectfully,

Susanna Valanne, Tiina S. Salminen, Mirva Järvelä-Stölting, Laura Vesala, Mika Rämet
